# Overall survival of pancreatic ductal adenocarcinoma is doubled by *Aldh7a1* deletion in the KPC mouse

**DOI:** 10.7150/thno.53935

**Published:** 2021-01-19

**Authors:** Jae-Seon Lee, Ho Lee, Sang Myung Woo, Hyonchol Jang, Yoon Jeon, Hee Yeon Kim, Jaewhan Song, Woo Jin Lee, Eun Kyung Hong, Sang-Jae Park, Sung-Sik Han, Soo-Youl Kim

**Affiliations:** 1 Division of Cancer Biology, Research Institute, National Cancer Center, Goyang, Republic of Korea.; 2 Department of Biochemistry, College of Life Science and Biotechnology, Yonsei University, Seoul, Republic of Korea.; 3 Graduate School of Cancer Science and Policy, National Cancer Center, Goyang, Republic of Korea.; 4 Division of Tumor Immunology, Research Institute, National Cancer Center, Goyang, Republic of Korea.; 5 Center for Liver and Pancreatobiliary Cancer, National Cancer Center, Goyang, Republic of Korea.; 6 Department of Pathology, National Cancer Center, Goyang, Republic of Korea.; 7 Department of Surgery, Center for Liver and Pancreatobiliary Cancer, National Cancer Center, Goyang, Republic of Korea.

**Keywords:** cancer metabolism, pancreatic ductal adenocarcinoma, ALDH7A1, oxidative phosphorylation complex I, KPC mice model

## Abstract

**Rationale:** The activity of aldehyde dehydrogenase 7A1 (ALDH7A1), an enzyme that catalyzes the lipid peroxidation of fatty aldehydes was found to be upregulated in pancreatic ductal adenocarcinoma (PDAC). *ALDH7A1* knockdown significantly reduced tumor formation in PDAC. We raised a question how ALDH7A1 contributes to cancer progression.

**Methods:** To answer the question, the role of ALDH7A1 in energy metabolism was investigated by knocking down and knockdown gene in mouse model, because the role of ALDH7A1 has been reported as a catabolic enzyme catalyzing fatty aldehyde from lipid peroxidation to fatty acid. Oxygen consumption rate (OCR), ATP production, mitochondrial membrane potential, proliferation assay and immunoblotting were performed. In *in vivo* study, two human PDAC cell lines were used for pre-clinical xenograft model as well as spontaneous PDAC model of KPC mice was also employed for anti-cancer therapeutic effect.

**Results:** *ALDH7A1* knockdown significantly reduced tumor formation with reduction of OCR and ATP production, which was inversely correlated with increase of 4-hydroxynonenal. This implies that ALDH7A1 is critical to process fatty aldehydes from lipid peroxidation. Overall survival of PDAC is doubled by cross breeding of KPC (*Kras^G12D^; Trp53^R172H^; Pdx1-Cre*) and *Aldh7a1^-/-^* mice.

**Conclusion:** Inhibitions of ALDH7A1 and oxidative phosphorylation using gossypol and phenformin resulted in a regression of tumor formation in xenograft mice model and KPC mice model.

## Introduction

Pancreatic ductal adenocarcinoma (PDAC) is known as a fatal cancer due to aggressively invasive and treatment-resistant malignancy. For decades, despite extensive efforts and the considerable progress made toward improving its detection 1, 2 and survival rates 3, 4, the 5-year survival rate for PDAC has not changed significantly 5, 6. The most common type of pancreatic cancer (~95%) is adenocarcinoma (PDAC). Although the main effective treatment is surgical resection followed by radiation and/or chemotherapy, over 80% of PDAC patients suffer from a progressive metastatic tumor that is unresectable by the time of diagnosis 7. Current therapeutic recommendation of PDAC was announced in American Society of Clinical Oncology Clinical Practice Guideline, which is FOLFIRINOX (leucovorin, fluorouracil, irinotecan, and oxaliplatin; favorable comorbidity profile) or gemcitabine plus nanoparticle albumin-bound (NAB) -paclitaxel 8.

Recently, people tried to find therapeutic possibility with immunotherapies including check point inhibitors, therapeutic vaccines, adaptive T cell therapy, and monoclonal antibodies against signaling molecules. However, any single agent of immunotherapies in pancreatic cancer does not improve overall survival significantly 9, 10. It may not be sufficient to remove the inhibitory immune regulators or to active innate and cytotoxic immunity alone in PDAC.

Among other approaches for finding effective therapeutics, progresses are noticed in cancer metabolism field. The first anti-cancer drug targeting metabolic abnormality in glioblastoma related with mutation of isocitrate dehydrogenase 2 has been approved from FDA 11. It is known that oncogene or cancer driver gene regulates cancer metabolism. With a near 100% *KRAS* mutation frequency, PDAC is considered the most RAS-addicted of all cancers 12. Therefore, PDAC shows RAS depended metabolic profiles, which accelerates glycolysis by induction of *GLUT1, HK1, PFK1* and *LDHA* as well as pentose phosphate pathway by induction of *RPE* and *RPIA* 13, 14. Glycolysis and pentose phosphate pathway are responsible metabolic pathway for building biomaterials such as nucleotide, amino acids, and fatty acids. However, there is no direct evidence that KRAS regulates ATP production in cancer. Among possible metabolic pathway of ATP production in PDAC, fatty acids and lipids in regulating the progression of PDAC has been observed. Lipids can stimulate proliferation of pancreatic cell lines, but did not stimulate growth of non-pancreatic cell lines 15. Pancreatic normal cells uses lipids as an energy source at higher rate than other tissue cell types 16. However, there is no investigation whether PDAC inherits physiological character of energy supply from normal pancreatic cells as fully equipped with machineries of lipid catabolism.

## Results

### *ALDH7A1* knockdown effectively reduced tumor growth with mitochondrial activity in PDAC

**Figure 1:**
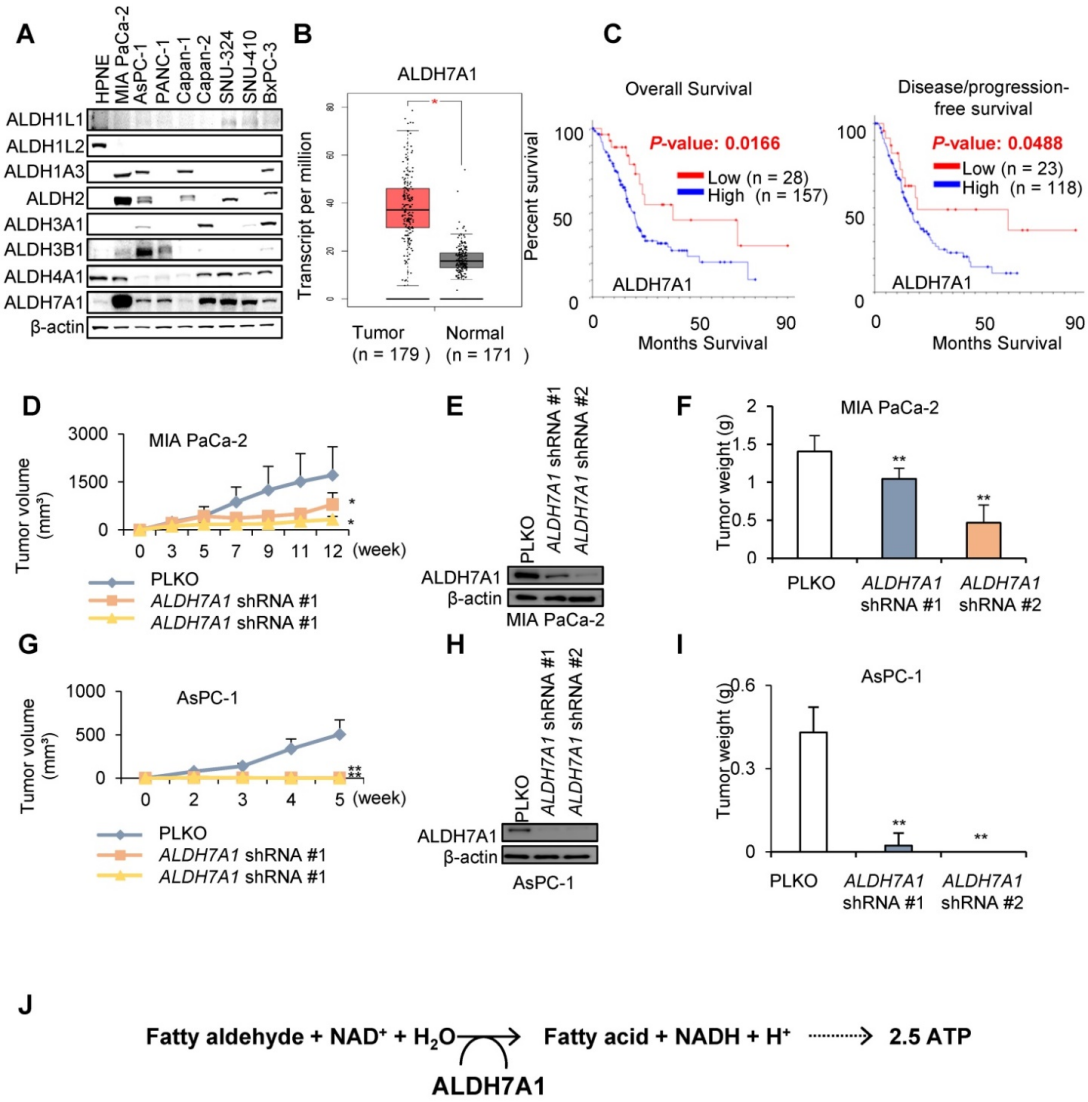
** ALDH7A1 is highly increased in PDAC. (A)** Western blot showed increased expression of ALDH7A1 in Pancreatic cancer cell lines compared to other ALDH isotypes. **(B)** ALDH7A1 expression level in PAAD patients was compared with matched normal by GEPIA webserver (http://gepia.cancer-pku.cn/). **(C)** PAAD patients with high ALDH7A1 expression showed poor prognosis than the others. Pancreatic adenocarcinoma (TCGA, Provisional) datasets were analyzed by cBioPortal (www.cbioportal.org). ALDH7A1 expression level less than standard deviation from the mean value was considered as low. **(D)** Knockdown of *ALDH7A1* suppressed tumor growth in MIA PaCa-2 cells xenograft mouse model. Graph shows a decrease in tumor growth as measured using calipers. **(E)** Western blot analysis of MIA PaCa-2 cancer cell lines demonstrating their ALDH7A1 status. Actin used as a loading control. **(F)** Final weight of subcutaneous tumors derived from MIA PaCa-2. **(G)** Knockdown of *ALDH7A1* suppressed tumor growth in AsPC-1 cells xenograft mouse model. Graph shows a decrease in tumor growth as measured using calipers. **(H)** Western blot analysis of AsPC-1 cancer cell lines demonstrating their ALDH7A1 status. Actin used as a loading control. **(I)** Final weight of subcutaneous tumors derived from AsPC-1. **(J)** Role of the ALDH7A1 in PDAC. *p < 0.05, **p < 0.01, ***p < 0.001.

Previously we have reported that cytosolic NADH production is responsible for ATP production in cancer cells through mitochondrial oxidative phosphorylation via malate aspartate shuttle (MAS) transportation of NADH in NSCLC and melanoma 17-20. Cytosolic ALDH produced major amount of NADH in the cytosol, which converted into ATP in cancer cells. Among 19 ALDH isoforms, eight ALDH isoforms (1A3, 1B1, 2, 3A1, 3B1, 4A1, 7A1, and 9A1) were upregulated in pancreatic cancer compared with matched normal by combined analysis of TCGA and GTEx data (Figure S1A and B). Among them only three isoforms (3A1, 3B1, and 7A1) were associated with poor prognosis of pancreatic cancer patients by analysis of TCGA data (Figure S1B). Of these three ALDH isoforms, ALDH7A1 is the most abundant ALDH isotype in PDAC cell lines in Western blotting (Figure 1A). Although the mRNA level of *ALDH7A1* is associated with poor survival of pancreatic cancer patients (Figure 1A and B, Figure S1C and D), further investigation may reveal protein level of ALDH7A1 and expression cell types. To test whether *ALDH7A1* is indeed highly upregulated in PDAC tumors to compare to the normal pancreas tissues, ALDH7A1 expression was observed in the tumors of KPC mouse to compare to normal pancreas of mouse. The staining showed significant increase of ALDH7A1 in the tumor of KPC mouse to compare to pancreas of normal mouse (Figure S1E).

To test whether ALDH7A1 plays an important role of tumor growth, PDAC cell lines with *ALDH7A1* knockdown were inoculated into the nude mouse and compared tumor growth with wild type cell lines (Figure 1D-I and Figure S1F and G*).* MIA PaCa-2 cell line with *ALDH7A1* knockdown showed over 30% reduction of tumor growth (Figure 1D-F and Figure S1F and H) as well as AsPC-1 cell line with *ALDH7A1* knockdown showed complete loss of tumor growth (Figure 1G-I and Figure S1G). ALDH7A1 is catabolizing efficiently lipid peroxidation-derived aldehydes such as propanal, hexanal, malondialdehyde and 4-hydroxy-2-nonenal (4-HNE) 21, 22. Catalytic reaction of ALDH7A1 produces fatty acid and NADH from fatty aldehyde (Figure 1J).

### ALDH7A1 is responsible for initiating step of fatty acid oxidation

Oxygen consumption rate (OCR) of PDAC cell lines were higher than that of normal HPNE (Human Pancreatic Nestin Expressing) cells by 1.8 ~ 6-fold (Figure S2A). Mitochondrial activity was higher than that of normal HPNE by 1.8 ~ 3.4-fold (Figure S2B). This implies that PDAC cell lines use more oxygen to operate mitochondrial oxidative phosphorylation (OxPhos) than normal cell does under normal culture condition.

**Figure 2:**
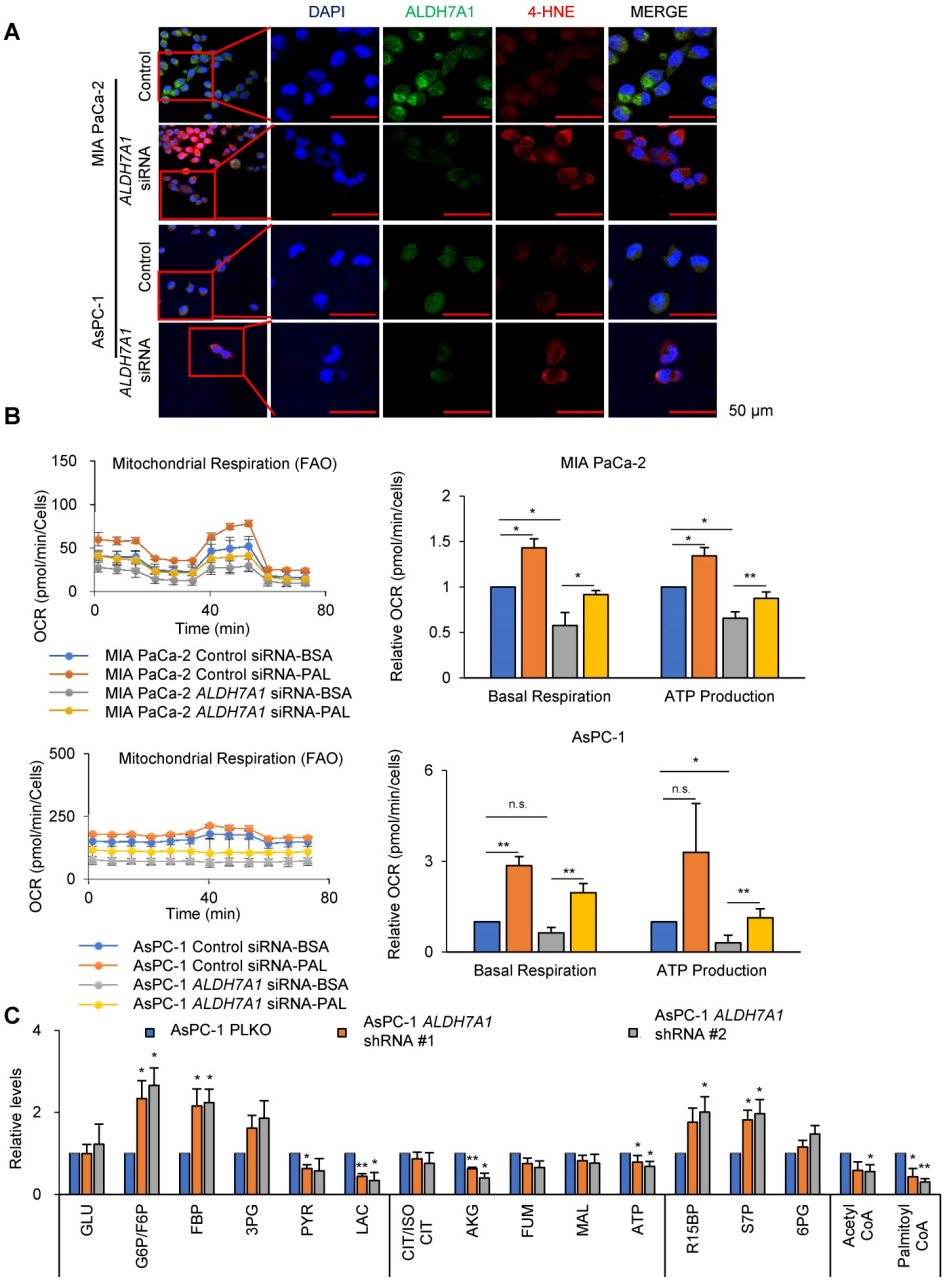
***ALDH7A1* knockdown induces 4-HNE accumulation and decrease in β-oxidation level. (A)** *ALDH7A1* knockdown increased 4-hydroxynonenal level in Pancreatic cancer cells as determined by Immunocytochemistry analysis. Scale bar = 50 µm. **(B)** Seahorse XF analysis of cells treated sequentially with oligomycin, the chemical uncoupler FCCP and antimycin A in the presence of bovine serum albumin alone (BSA) or palmitate-BSA. *ALDH7A1* knockdown cells showed decreased fatty acid oxidation compared to control cells. **(C)** Effect of *ALDH7A1* siRNA treatment (40 nM for 48 h) on metabolites derived from various metabolic pathways in AsPC-1. Data are expressed as the mean and standard deviation of three independent experiments. *p < 0.05, **p < 0.01, ***p < 0.001.

ALDH7A1 catalyzes 4-HNE to 4-hydroxy-2-nonenoic acid (4-HNA) and NADH, which further catalyzes to acyl-CoA for fatty acid oxidation 23. We have tested whether *ALDH7A1* knockdown increases the level of 4-HNE in PDAC cell lines (Figure 2A). MIA PaCa-2 and AsPC-1 showed increase of 4-HNE level by siRNAs of *ALDH7A1* treatment by immunohistochemical staining of 4-HNE (Figure 2A). To test whether ALDH7A1 activity is required for fatty acid oxidation in PDAC cells, fatty acid oxidation (FAO) analysis was performed by OCR measurement (Figure 2B). After transfection of *ALDH7A1* siRNAs for 48 h, cells were cultured under substrate-limited medium with 0.5 mM glucose and 1% FBS for 24 h at 37 ℃. The next day, the medium was changed to assay medium and OCR analyzed with treatment of palmitate-BSA (palmitate conjugated with Bovine Serum Albumin). In the fatty acid supplement experiment, OCR implies the amount of fatty acid oxidation in cancer cells, which resulted in 37% and 132% increase of OCR concomitantly with increase of 30% and 140% increase of ATP by palmitate supplement in MIA PaCa-2 and AsPC-1 respectively (Figure 2B). Furthermore, *ALDH7A1* knockdown showed 36% and 56% decrease of ATP production in MIA PaCa-2 and AsPC-1 respectively (Figure 2B).

To test whether ALDH inhibitor gossypol also induces the same effect as *ALDH7A1* knockdown, 4-HNE level was analyzed by immunohistochemical staining after gossypol treatment for 24 h (Figure S2C). MIA PaCa-2 and AsPC-1 showed significant increase of 4-HNE level by gossypol treatment (Figure S2C). FAO analysis with gossypol treatment was performed by OCR measurement (Figure S2D). Gossypol treatment showed 94% and 83% decrease of ATP production in MIA PaCa-2 and AsPC-1 respectively (Figure S2D). In the fatty acid supplement experiment, OCR resulted in 32% and 140% increase of OCR concomitantly with increase of 43% and 208% increase of ATP by palmitate supplement in MIA PaCa-2 and AsPC-1 respectively (Figure S2D). To test whether ATP production depends on fatty acid oxidation through ALDH7A1, metabolite analysis was performed in cells subjected to ALDH7A1 knockdown for 24 h (Figure 2E). *ALDH7A1* knockdown in AsPC-1 cells had no significant reduce on metabolites derived from the glycolysis and pentose phosphate pathways. ATP production fell by approximately 30% after knockdown of *ALDH7A1* (Figure 2E), which was accompanied by a fall in levels of acetyl-CoA (Figure 2E). We also performed metabolite analysis in cells treated for 24 h with gossypol (Figure S2E). Gossypol acts as a reversible noncompetitive inhibitor of ALDHs. Gossypol may interact with the cofactor binding site. ALDH inhibition had no significant reduce on glycolysis and pentose phosphate pathway metabolites. ATP production fell by 50% (Figure S2E) and was accompanied by a reduction in a 90% reduction in acetyl-CoA (Figure S2E). This suggests that pancreatic cancer cells depend on fatty acids to generate acetyl-CoA through fatty aldehydes.

It is known that ROS level is higher in cancer cells which may triggers lipid peroxidation turning fatty acid to fatty aldehyde 23, 24. Therefore, PDAC cells may be adopted lipid peroxidation for generating fatty aldehyde using ROS and employed ALDH7A1 to produce NADH and fatty acid for β-oxidation.

### *ALDH7A1* knockdown induced growth arrest accompanied with OCR reduction

**Figure 3:**
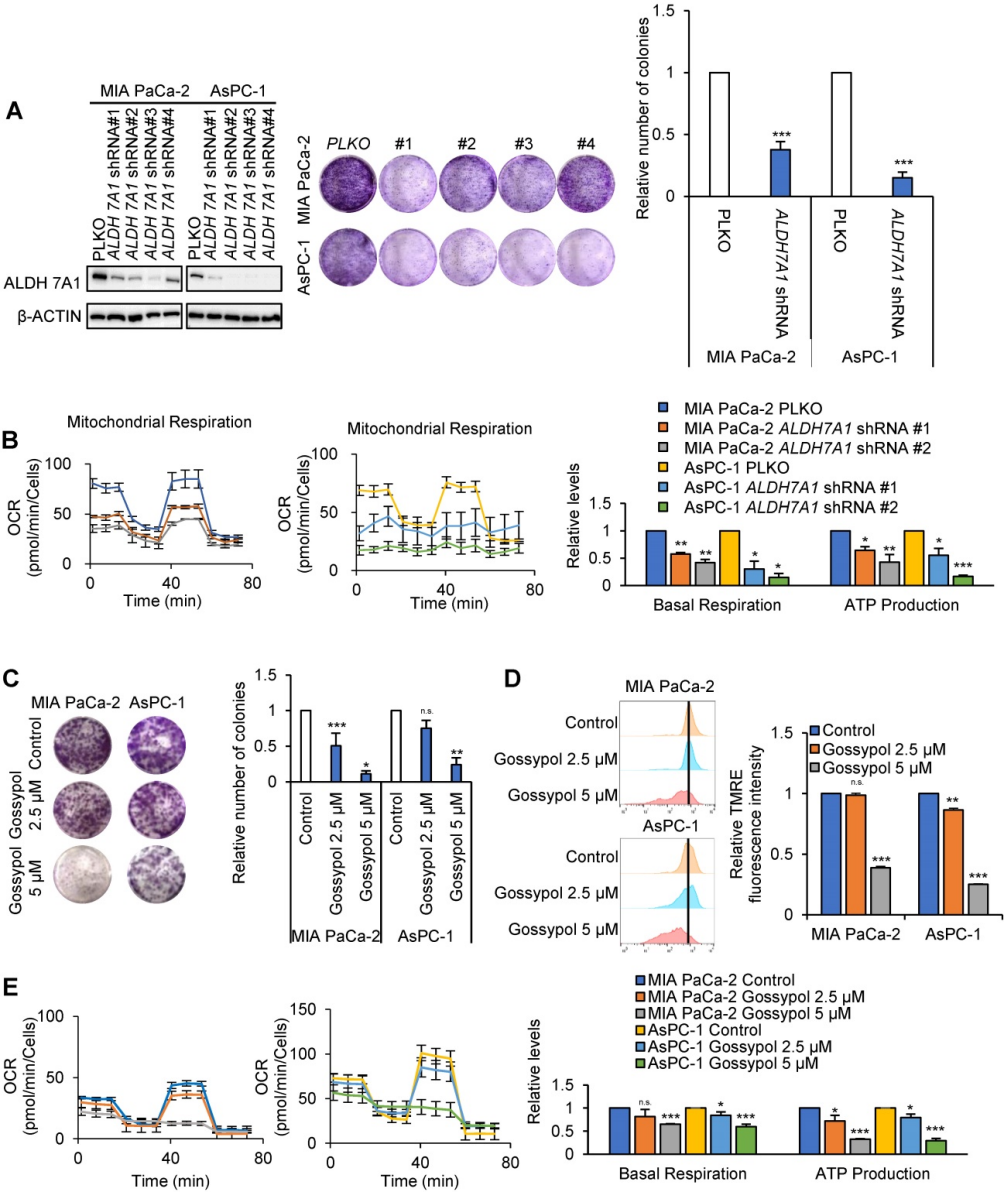
***ALDH7A1* knockdown suppressed proliferation of pancreatic cancer by reduced ATP production. (A)** The proliferation was analyzed by crystal violet cell colony assay in *ALDH7A1* knockdown pancreatic cancer cell lines in comparison with pLKO.1 pancreatic cancer cell line. **(B)** The oxygen consumption rate (OCR) was analyzed using the Seahorse XFe96 analyzer in pancreatic cancer cell lines compared to *ALDH7A1* knockdown pancreatic cancer cell lines and normalized by SRB assay. **(C)** Treatment of gossypol for 72 h showed inhibition of cell growth in pancreatic cancer cell, as determined by the clonogenic assay. **(D)** treatment of gossypol for 24 h reduced mitochondrial membrane potential, as determined by TMRE staining and FACS analyzer. **(E)** treatment of gossypol for 24 h reduced oxygen consumption rates (OCR) and respiration parameters as determined by Seahorse XFe96 analyzer. Data are expressed as the mean and standard deviation of three independent experiments. *p < 0.05, **p < 0.01, ***p < 0.001.

To test whether *ALDH7A1* knockdown may affect cancer cell growth, colony formation assays and OCR were performed (Figure 3A and B). Colony formation of pancreatic cancer cell lines were reduced average 64% and 85% by *ALDH7A1* knockdown in MIA PaCa-2 and AsPC-1 respectively (Figure 3A). OCR was reduced average 64% and 76% concomitantly with decrease of ATP production to 54% and 36% level of the control by *ALDH7A1* knockdown in MIA PaCa-2 and AsPC-1 respectively (Figure 3B). To test whether cell proliferation and oxygen consumption rate were recovered by re-expression of *ALDH7A1*, cell proliferation and OCR assay were performed. We observed that cell proliferation was recovered up to 1.77-fold to compare to *ALDH7A1* knockdown by *ALDH7A1* re-expression in an expression dependent manner (Figure S3A and B). Also, oxygen consumption rate and ATP production were recovered up to 1.46-fold and 1.41-fold to compare to *ALDH7A1* knockdown respectively (Figure S3C). This suggests that ALDH7A1 contributes to the increase of ATP production by 4-hydroxynonenoic acid for further oxidation and NADH production. In order to test whether ALDH inhibition using ALDH inhibitor gossypol also results in the same effect as *ALDH7A1* knockdown, colony formation assay, TMRE measurement and OCR assay were performed with treatment of gossypol in MIA PaCa-2 and AsPC-1 cell lines (Figure 3C-E). Colony formation of pancreatic cancer cell lines were reduced up to 90% and 75% by gossypol treatment in a dose dependent manner in MIA PaCa-2 and AsPC-1 respectively (Figure 3C). Mitochondrial activity measured by TMRE was decreased up to 62% and 75% by gossypol treatment in a dose dependent manner in MIA PaCa-2 and AsPC-1 respectively compared to the control (Figure 3D). The basal level of OCR was reduced up to 45% by gossypol treatment concomitantly with decrease of ATP production up to 25% level of the control by gossypol treatment in a dose dependent manner in both MIA PaCa-2 and AsPC-1 (Figure 3E).

### Combination treatment with gossypol and phenformin induces cancer cell death following ATP depletion

**Figure 4:**
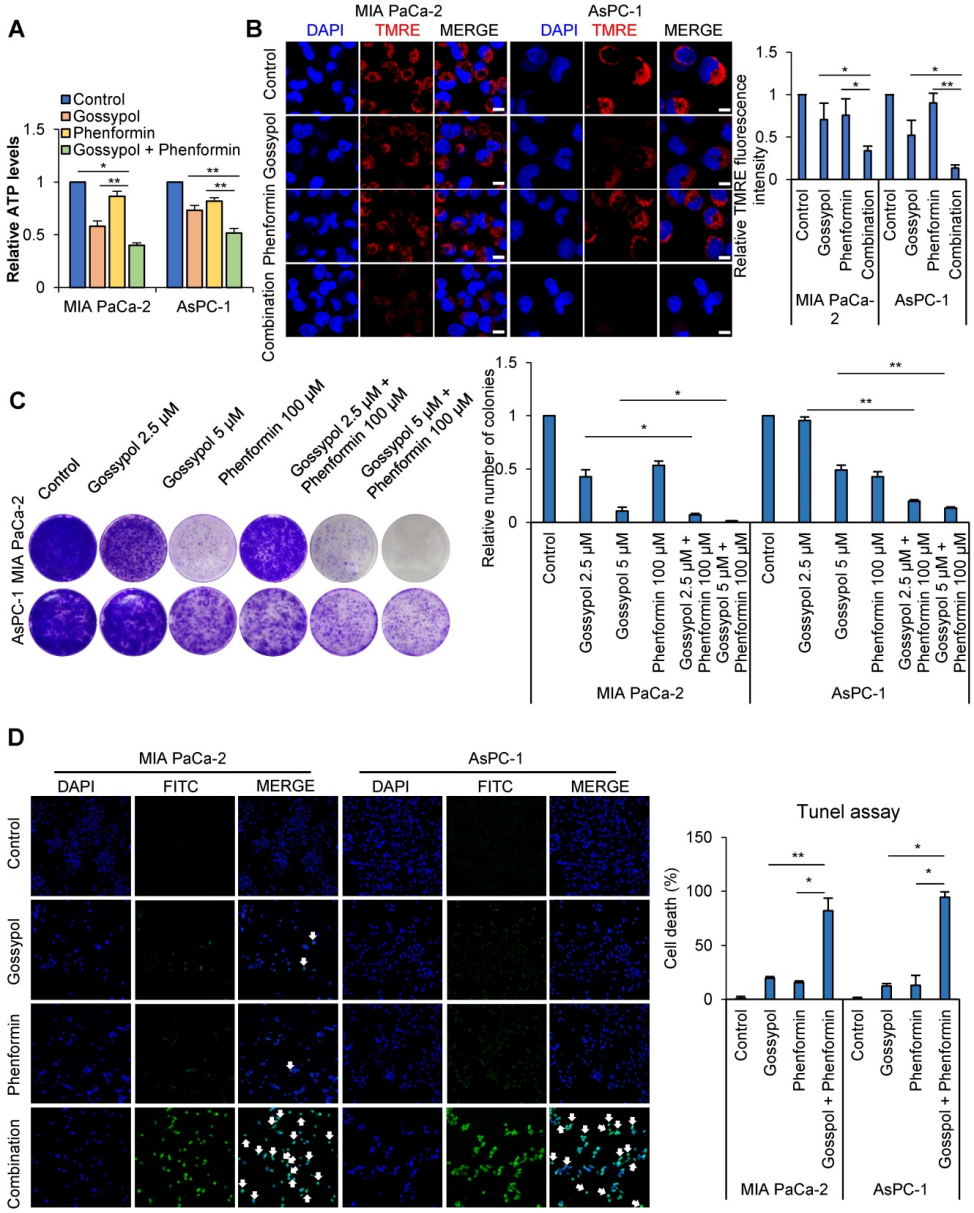
** Pancreas normal duct cell was not affected with combination treatment of gossypol and phenformin. (A)** Combined treatment for 24 h reduced ATP production synergistically, as determined by ATP colorimetric assay kit. **(B)** Combined treatment for 24 h reduced mitochondrial membrane potential synergistically, as determined by TMRE staining and confocal microscopy. Scale bar = 50 µm. **(C)** Combined treatment of 5 µM gossypol with 100 µM phenformin for 72 h showed synergistic inhibition of cell growth in pancreatic cancer, as determined by the clonogenic assay. **(D)** Synergistic effect of combined treatment of 5 µM gossypol and 100 µM phenformin after 48h on cell death was determined by TUNEL assay. Scale bar = 200 µm. Data are expressed as the mean and standard deviation of three independent experiments. *p < 0.05, **p < 0.01, ***p < 0.001.

Decrease in ATP level induced cell cycle arrest at G1/S transition as well as G2/M transition 25, 26. Previously delay in mitotic progression was shown by combined treatment of inhibitors against ALDH and mitochondria complex I accompanying synergistic decrease of ATP production 17-19. The combined treatment of gossypol and phenformin for 12 h reduced OCR to 50% and 66% levels compared to the control in MIA PaCa-2 and AsPC-1 respectively (Figure S4A). ATP production was reduced by combined treatment of gossypol and phenformin for 24 h to 32% and 51% levels (Figure 4A) concomitantly with decrease of mitochondrial membrane potential as 67% and 87% compared to the control in MIA PaCa-2 and AsPC-1 respectively (Figure 4B) whereas no changes were observed in HPNE cells (Figure S4B). To test whether decrease of ATP by combination of gossypol and phenformin reduce cancer growth, colony formation assay was performed. Colony formation was reduced up to 99% and 87% compared to the control by 72 h treatment in a dose dependent manner in MIA PaCa-2 and AsPC-1 respectively (Figure 4C). We have tested whether combined treatment of gossypol and phenformin induces cell death using DAPI and TUNEL (Figure 4D). By combination treatment of gossypol and phenformin for 48 h, cell death was induced in 81% and 94% cell populations of MIA PaCa-2 and AsPC-1 respectively while cell death was induced in 37% and 17% cell population of MIA PaCa-2 and AsPC-1 cells respectively by single treatment of gossypol (Figure 4D) whereas no cell death was observed in HPNE cells by single or combination treatment (Figure S4C). It is a critical difference between normal and cancer cells that only cancer cells induce cell death when ATP production is reduced.

### Loss of ALDH7A1 reduced tumor growth in preclinical PDAC models

**Figure 5:**
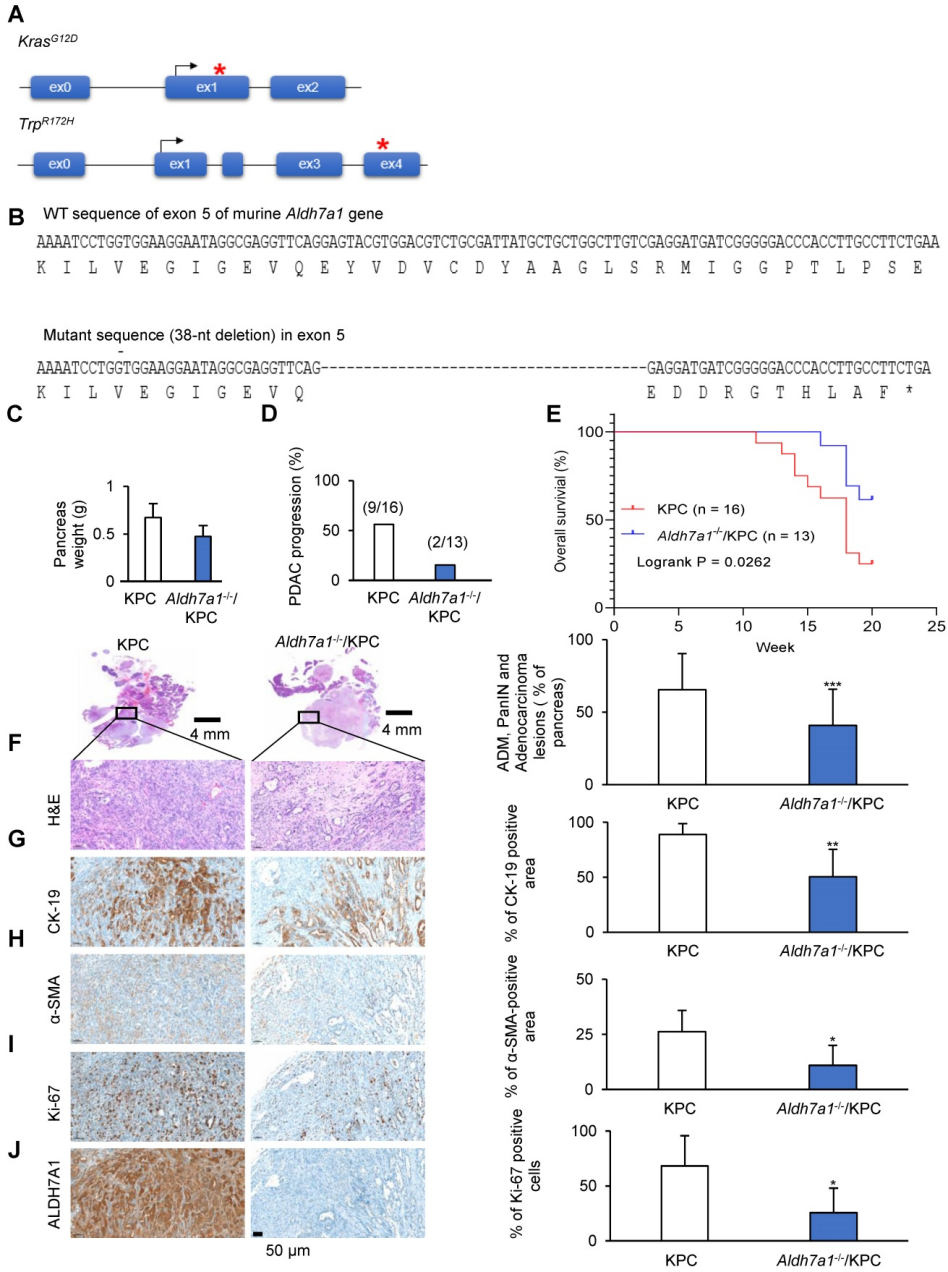
** ALDH7A1 deficiency causes a significant reduction in pancreatic cancer progression of mouse. (A)** In KPC mouse, the conditional expression of mutant *Kras^G12D^* and *Trp53^R172H^* is controlled by a *Pdx1-Cre*. **(B)** DNA sequence and peptide sequence of murine *Aldh7a1* knockout mouse having 38-nt deletion in exon 5, was used in breeding with KPC mouse. This frameshift mutation causes premature translation termination. **(C)** Quantification of pancreas weight in *Aldh7a1* knockout; KPC mouse and KPC mouse. **(D)** Frequency of pancreatic duct adenocarcinoma in *Aldh7a1* knockout; KPC mouse and KPC mouse. **(E)** Kaplan-Meier survival curves of KPC mouse and *Aldh7a1* knockout; KPC mouse. **(F-J)** H&E **(F)**, CK-19 **(G)**, α-SMA **(H)**, Ki-67 **(I)** and ALDH7A1 **(J)** staining of the pancreas *Aldh7a1* knockout; KPC mouse and KPC mouse. And quantification of the percentage of lesions, CK-19, α-SMA and Ki-67 positive area. Scale bar = 50 µm. *p < 0.05, **p < 0.01, ***p < 0.001.

A transgenic mouse model has been created that expresses physiological levels of oncogenic Kras (*Kras^G12D^*) and mutant Trp53 (*Trp53^R172H^*) in the progenitor cells of mouse pancreas, which is KPC mouse model (*Kras^G12D^; Trp53^R172H^; Pdx1-Cre*). The KPC mice develop the full spectrum of pancreatic ductal adenocarcinoma from preinvasive neoplasias (PanINs) to invasive and metastatic disease (Figure 5A).

To investigate the role of ALDH7A1 in PDAC, *Aldh7a1* knockout mouse has been generated using CRIPSR/Cas9 genome editing described in Material and Methods or Supplementary Data. To generate knockout mouse having indel mutation in exon 5 of murine *Aldh7a1* gene, target sequences of sgRNA were selected using CRISPR design tool (crispor.tefor.net) and indel mutations in F1 mice were identified using Sanger sequencing. The mice having 38-nt deletion was used for further breeding because the mutation causes premature translation stop (Figure 5B, Figure S5A). Homozygote mutant of *Aldh7a1* showed normal phenotype in mice, which is concord with the previous report (http://www.informatics.jax.org/marker/MGI:108186). The weight of pancreas was significantly decreased in *Aldh7a1*^-/-^; KPC mouse compared with KPC mice (Figure 5C). H&E-stained pancreas histology images suggested that *Aldh7a1* knockout remarkably suppressed the progression of pancreatic ductal adenocarcinoma (Figure 5D*,* Figure S5B). Survival time of *Aldh7a1*^-/-^; KPC mice was also significantly increased than KPC mice (Figure 5E). And acinar-duct metaplasia (ADM), PanIN and pancreatic duct adenocarcinoma lesions of *Aldh7a1*^-/-^; KPC mice was reduced approximately 40% compared to the KPC mice (Figure 5F)*.* Cytokeratin-19 (CK-19) expression, a ductal epithelial marker, was also reduced 44% in *Aldh7a1*^-/-^; KPC mice than KPC mice (Figure 5G)*.* Pancreatic cancer in KPC mice has been known to be a highly aggressive malignancy with a prominent desmoplastic stroma which could be analyzed by α-smooth muscle actin (α-SMA) staining. Immunohistochemistry for α-SMA provided clear evidence that desmoplasia or stroma fibrosis, in particular fibroblast density 27. We observed that α-SMA-positive area of *Aldh7a1*^-/-^; KPC pancreas compared with KPC pancreas were reduced 62% (Figure 5H). Ki-67 positive cell (proliferation marker) of pancreas in KPC mice showed that *Aldh7a1* knockout decreased proliferation of cancer cell (Figure 5I). ALDH7A1 was not expressed in *Aldh7a1*^-/-^; KPC (Figure 5J)*.* Taken together, these results suggest that ALDH7A1 deficiency causes a significant reduction in pancreatic cancer progression of mice, implying the critical role of ALDH7A1 in PDAC.

### Combined inhibition of ALDH7A1 and mitochondrial complex I synergistically abrogated tumor growth in preclinical PDAC models and KPC mouse models

**Figure 6:**
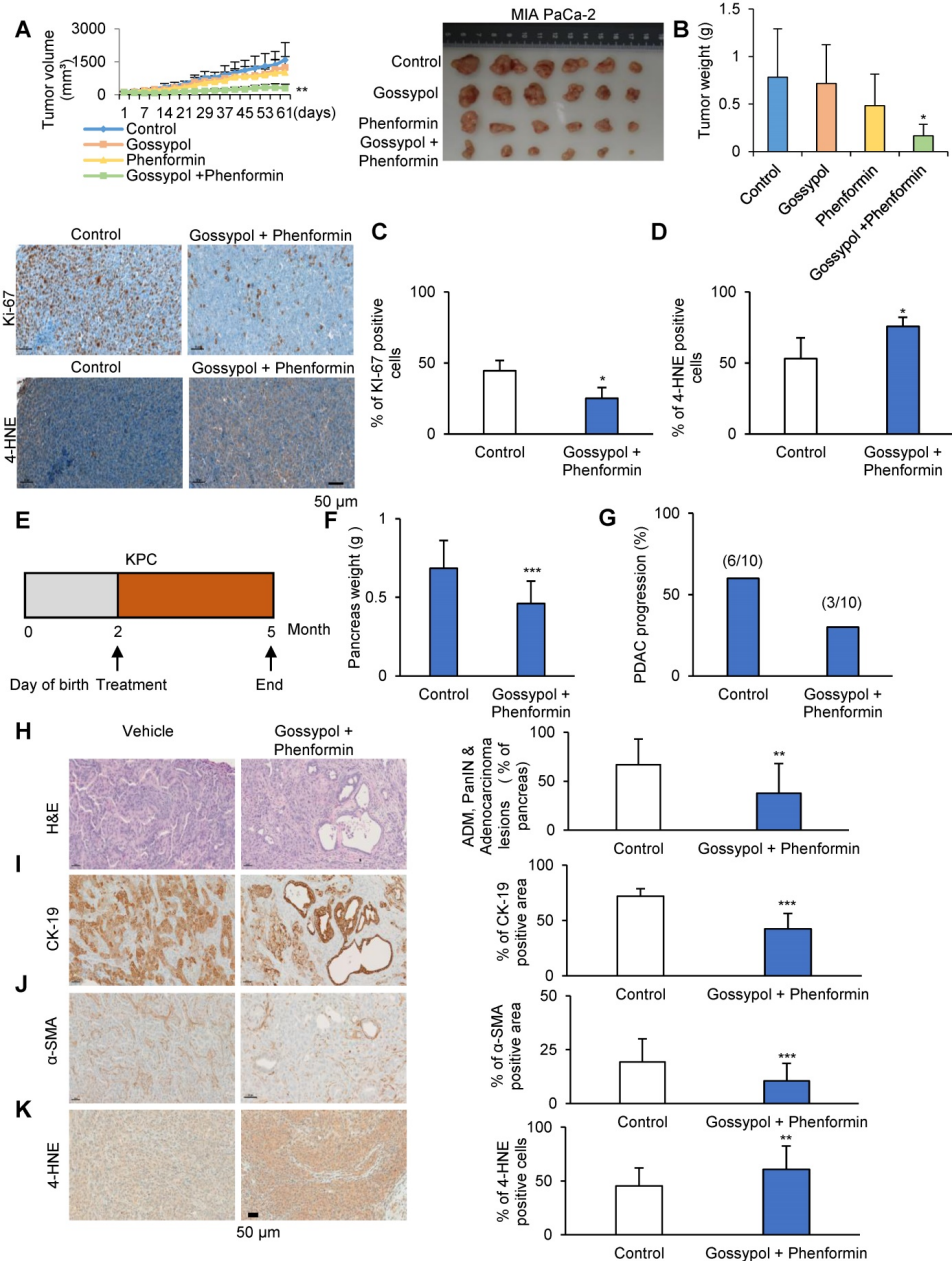
** Gossypol treatment combined with phenformin synergistically suppresses tumor growth in a human pancreatic cancer xenograft mouse model and KPC mouse model. (A)** MIA PaCa-2 (1 × 10^7^) cells were injected in 6-8-week-old BALB/c nude mice. When the volume of the tumor mass reached 110 mm^3^, the mice were randomly assigned to one of four treatment groups including vehicle control, gossypol, phenformin, and combination of gossypol and phenformin (n=6 per group). Gossypol (40 mg/kg body weight), phenformin (100 mg/kg body weight), and vehicle were administered orally 6 days/week. Graph (left) and photograph (right) shows a synergistic decrease in tumor growth after combined treatment of gossypol and phenformin as measured using calipers. **(B)** Final weight of subcutaneous tumors derived from MIA PaCa-2. **(C)** IHC analysis of Ki-67 staining in MIA PaCa-2 tumor xenograft tissues. Scale bar = 50 µm. **(D)** IHC analysis of 4-HNE staining in MIA PaCa-2 tumor xenograft tissues. Scale bar = 50 µm. **(E)** Scheme showing the experimental design of drug treatment protocols in KPC mouse **(F)** Quantification of pancreas weight in mice treated with vehicle or gossypol combined with phenformin. **(G)** Frequency of pancreatic duct adenocarcinoma in KPC mouse treated with vehicle or gossypol combined with phenformin. **(H-K)** H&E (H), CK-19 (I), α-SMA (J) and 4-HNE (K) staining of the pancreas in vehicle or gossypol combined with phenformin-treated mouse and quantification of the percentage of CK-19, α-SMA and 4-HNE positive area in mouse treated with vehicle or gossypol combined with phenformin. Scale bar = 50 µm. *p < 0.05, **p < 0.01, ***p < 0.001.

We tested whether combination treatment with gossypol and phenformin produced any synergistic therapeutic effect in the PDAC mouse xenograft model using MIA PaCa-2 (Figure 6A-D) or AsPC-1 cells (Figure S6A-D). Cultured MIA PaCa-2 or AsPC-1 cells were injected subcutaneously near the scapulae of 8-week-old female nude BALB/c mouse. Oral administration of gossypol (MIA PaCa-2: 40 mg/kg; AsPC-1: 80 mg/kg), phenformin (100 mg/kg), and gossypol (MIA PaCa-2: 40 mg/kg; AsPC-1: 80 mg/kg) combined with phenformin (100 mg/kg) was initiated when tumors reached a volume of 100 mm^3^ and was continued for 6 days per week. Body weight change over 10% compared to the control was not observed in mouse that received the combination treatment for 2 weeks (Figure S6E and F). Single administration of gossypol did not show any therapeutic efficacy in mouse (Figure 6A-D and Figure S6A-D) while gossypol single treatment showed decrease of colony formation *in vitro* culture system (Figure 3C and 4C). However, combined treatment of gossypol and phenformin showed 80% decrease of tumor weight in MIA PaCa-2 (Figure 6A and B) and 52% decrease of tumor weight in AcPC-1 (Figure S6A and B) as *in vitro* culture also showed synergistic effect of gossypol and phenformin (Figure 4C). Tumors were collected at the end of the study and immunohistochemical staining for Ki-67 and 4-HNE was performed (Figure 6C and D, Figure S6C and D). Ki-67 staining showed a clear inverse correlation with combination treatment (Figure 6C and Figure S6C). MIA PaCa-2 and AsPC-1 xenograft tissues showed significant increase of 4-HNE level by Combination treatment (Figure 6D and Figure S6D). After treatment, tumor volumes were reduced significantly with combination therapy compared to vehicle-treated control as well as single drug-treated groups while body weight remained constant, clearly demonstrating the enhanced efficacy of combined treatment *in vivo*. To evaluate the therapeutic effect of gossypol combined with phenformin in pancreatic cancer treatment, we assessed whether treatment with concomitant drug suppress tumor progression in KPC mice model (Figure 5A). Starting at 2 months of age, KPC mice were treated daily with gossypol combined with phenformin or vehicle for 3 months (Figure 6E). Tumor volume and weight were decreased as well as adenocarcinoma progression was also reduced by combined treatment of gossypol and phenformin (Figure 6F and G, Figure S6G and Figure S7). Overall survival of KPC mice showed superior difference in the group of combined treatment (Figure S6H). A histological examination of adenocarcinoma progression was analyzed by Hematoxylin and Eosin staining and immunohistochemical staining of CK-19, α-SMA, and 4-HNE (Figure 6H-K). CK-19 is expressed specifically in the duct cells of pancreas, which is known as a marker of PDAC 28. α-SMA is a well-characterized marker of activated pancreatic stellate cells (PSCs) 27. Therefore, the progression of pancreatic cancer (PanIN or PDAC) in KPC has been analyzed by immunostaining of CK-19 and α-SMA. Lesions in which H&E-stained pancreas tissue image suggests that the combination of gossypol and phenformin approximately 45% inhibited the progression of pancreatic adenocarcinoma, ADM, and pancreatic duct adenocarcinoma compared to vehicle treatment (Figure 6H). CK-19 expression was also reduced 40% in combination treatment mice than vehicle treatment mice (Figure 6I). We observed that α-SMA-positive area of combination treatment mice pancreas compared with vehicle treatment mice pancreas were reduced 48% (Figure 6J). KPC mice pancreas tissues showed significant increase of 4-HNE level by Combination treatment (Figure 6K). After treatment, tumor progression were reduced significantly with combination therapy compared to vehicle-treated control as well as single drug-treated groups while body weight remained constant, clearly demonstrating the enhanced efficacy of combined treatment *in vivo*.

## Discussion

It is astonishing finding that ATP production has not been changed by glucose depletion for 24 h in PDAC cell lines under normal culture condition 29. This implies that ATP production of PDAC does not depend on glycolysis but depends on oxidative phosphorylation through fatty acid oxidation. *ALDH7A1* knockdown showed increase of fatty aldehyde HNE level which is inversely correlated with significant decrease of ATP production. Here, by targeting ATP production through inhibition of ALDH7A1 and mitochondrial complex I, remarkable regression of tumor size and number in xenograft model as well as KPC mouse model based on *Kras* mutation has been shown. In the connecting molecular mechanism between ATP depletion and tumor regression, mTOR was the key master regulated by ATP level that command biogenesis, adipogenesis, and angiogenesis. mTOR is a ATP sensor itself 30 although mTOR is known to be regulated by many cellular signals including growth factors, insulin, glucose, amino acids, AMPK and hypoxia 31. In this study, growth arrest by ATP depletion was induced by mTOR inactivation, which later lost control of survival. This suggests possibility of therapeutic approach that targets specifically cancer without any harm to normal cells, because normal cell depends on TCA cycle for ATP production using glucose.

Warburg found that cancer cells produce lactate instead of CO_2_ using glucose. He misunderstood that cancer cells should rely on the glycolysis as a major source of ATP production due to the destruction of mitochondria. The idea of “broken mitochondria” prevented understanding active OxPhos in cancer cell catabolism 32-35. Recent study showed that mitochondria in cancer cells are intact, and cancer cells have a higher respiratory rate than normal cells 29, 36, 37. Indeed, cancer cells depend on mitochondrial OxPhos for ATP production 29. According to a report on the contribution of the pathways to ATP in cancer cells, OxPhos is responsible for about 90% of ATP production despite the increase of the glycolysis 38-40. The transfer of electrons from NADH or FADH_2_ to O_2_ by a series of electron carriers in OxPhos results in the ATP production. Therefore, OxPhos is active for ATP synthesis in cancer cells while glucose is used in glycolysis for carbon sources. Recently we found that major source of NADH and FADH_2_ is cytosolic production which is transferred into mitochondria either through MAS for NADH transfer or mid-chain fatty acid for fatty acid oxidation 29. The NADH transferred either thorough MAS or MCFA drives strong electron-transport activity.

Results of this study imply that ALDH7A1 deficiency in pancreatic cells delays the progression of PDAC in KPC mice with following reasons. First, expression of oncogenic Kras and p53 is restricted in pancreas, especially Pdx1-Cre expressing cells because *Pdx1-Cre* is active in the pancreas by E9.5, in the pancreatic epithelium, and beta islet cells in adults 41. Second, mice homozygous for disruptions in *Aldh7a1* gene has been known to display a normal phenotype (http://www.informatics.jax.org/allele/MGI:3530146). Third, our results with cancer cell lines and xenograft models also suggest that knockout and knockdown in pancreas cells has an inhibitory effect on the progression of pancreatic cancer. Taken together, ALDH7A1 deficiency in pancreatic cells appears to delay the progression of PDAC in KPC mice. However, role of ALDH7A1 in pancreatic stellate cell may be also important because stellate cell plays a role as a cancer associate fibroblast. The role of ALDH7A1 in stellate cancer associate fibroblast using knock out mice will be investigated in the next study.

Several roles of ALDH7A1 have been reported. ALDH7A1 plays also protecting against hyperosmotic stress by generating betaine as an osmolyte through catalyzing betaine aldehyde 42. ALDH7A1 also involves in lysine catabolism by catalyzing aminoadipic semialdehyde into aminoadipic acid for maintaining cellular nitrogen pools and also serves as a source for ketone bodies as an important energy source in the heart and brain 42. However, betaines are widely distributed in microorganisms, plants, and animals as well as ketone bodies are abundantly produced from fatty acid. Therefore, it is reasonable to consider that anti-cancer effect of *ALDH7A1* knockdown or inhibition resides on blocking fatty acid oxidation through inhibition of fatty aldehyde catalysis because level of fatty aldehyde (HNE) is inversely correlated with ATP production.

In PDAC, over 90% of pancreatic cancers showed about 100% frequency of *KRAS* mutation 13. Recently, RAS mediated changes in cell metabolism have been investigated whether these changes could be exploited for new therapeutic approaches. They are focused on anabolic metabolism including glycolysis, glutaminolysis, and pentose phosphate pathway. The most important discovery in this study is that PDAC shows higher OCR activity compared to the normal cell lines as well as PDAC produces more ATPs compared to the control cells under normal culture condition. In addition to that, ATPs production depends on fatty acid oxidation instead of TCA cycle in PDAC. Another differentiated phenomenon from normal cells is that fatty acid oxidation mostly occurs at peroxisome using ROS instead of mitochondrial β-oxidation. Therefore, the relation between KRAS mutation and fatty acid β-oxidation in PDAC remains to be clarified in near future.

**Figure 7:**
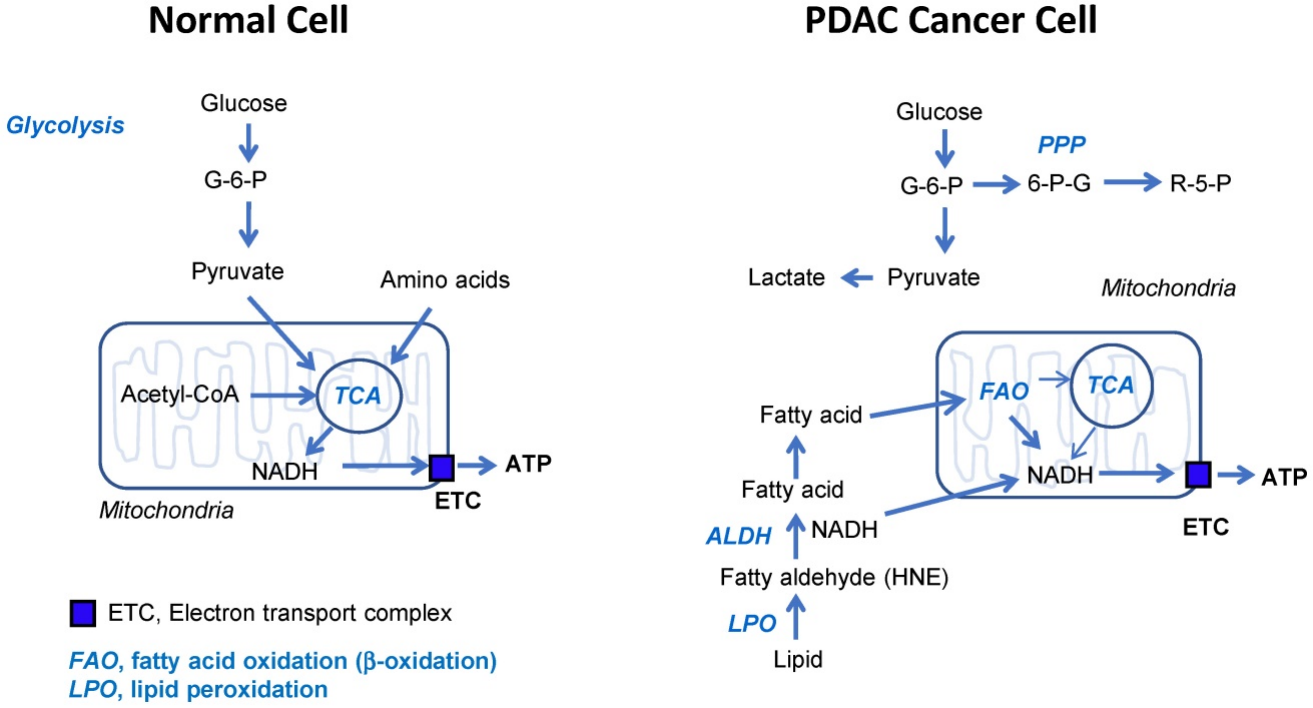
** Schematic diagram of normal and cancer energy metabolism.** This is based on the relative contribution to ATP production. The most important things are; 1) glucose is not ATP source in cancer, 2) fatty acid oxidation is the most dependent ATP source in cancer, 3) OxPhos is active in cancer. Therefore, targeting ALDH7A1 and mitochondrial complex I selectively blocks cancer energy metabolism.

It is reported that mitochondrial metabolism and ROS generation are essential for Kras-mediated tumorigenicity 43. Lipid peroxidation is an autocatalytic process initiated by ROS attack on the unsaturated fatty acids. The resulting lipid radicals rapidly interacts with oxygen, thereby propagating the reaction via peroxyl radical intermediates such as fatty aldehydes 44. One specific product of lipid peroxidation called 4-HNE is recognized as particularly important mediator of cellular function 44. ALDH7A1 connects between lipid peroxidation and β-oxidation for further catabolic process to increase mitochondrial oxidative phosphorylation (Figure 7). Therefore, phenformin combination with gossypol achieved synergistic depletion of ATP production by targeting NADH production and OxPhos activity respectively.

In conclusion, over expression of ALDH7A1 was related with bad overall survival in PDAC patients. By cross breeding of* Aldh7a1*^-/-^ mice and KPC (*Kras^G12D^; Trp53^R172H^; Pdx1-Cre*) and model, overall survival of PDAC was doubled. Furthermore, by blocking ALDH7A1 and mitochondrial complex I using gossypol and phenformin, tumor regression was synergistically induced with reduction of ATP level in mice models.

## Materials and methods

### Cell culture studies

AsPC-1, BxPC-3 and SNU-213 cells were grown in RPMI 1640 medium (SH30027.01, HyClone, Logan, UT, USA) containing 10% fetal bovine serum (SH30070.03HI, HyClone, Logan, UT, USA) and Cellmaxin (C3314-020, GenDEPOT, Texas, USA). SNU-324 cells were grown in RPMI 1640 medium containing 20% fetal bovine serum and Cellmaxin. MIA PaCa-2 and Panc-1 cells were grown in DMEM/High glucose medium (SH30243.01, HyClone, Logan, UT, USA) containing 10% fetal bovine serum and Cellmaxin. Capan-1 cells were grown in IMDM (12440053, Gibco, Logan, UT, USA) containing 20% fetal bovine serum and Cellmaxin. Capan-2 cells were grown in McCoy's 5A (16600082, Gibco, Logan, UT, USA) containing 10% fetal bovine serum and penicillin. hTERT-HPNE cells were grown in 75% DMEM without glucose (D-5030, Sigma-Aldrich, St. Louis, MO, USA) with additional 2 mM L-glutamine and 1.5 g/L sodium bicarbonate, 25% Medium M3 Base (Incell Corp. Texas, USA) containing 5% fetal bovine serum, 5.5 mM D-glucose (G8270, Sigma-Aldrich, St. Louis, MO, USA), Cellmaxin, 10 ng/ml human recombinant EGF (E9644, Sigma-Aldrich, St. Louis, MO, USA). Cells were incubated at 37 °C and maintained at 5% CO_2_. siRNA duplexes targeting human *ALDH7A1* were transfected into cells for 48 h using Lipofector-Q Reagent (AB-LF-Q001, AptaBio, Yongin, Korea) and Plusfector Reagent (AB-PF-0001, AptaBio, Yongin, Korea) according to the manufacturer's instructions. The siRNA sequences and overexpression primer of *ALDH7A1* are listed in Table S1 and 2.

### Construction of stably transfected cell lines

*ALDH7A1* shRNA plasmids were purchased from Sigma-Aldrich (SHCLNG-NM_001182). Lentivirus packaging was in 293T cells. Following viral infection and puromycin screening, stably transfected cells were acquired. The shRNA sequences are listed in Table S3.

### Immunocytochemistry

For immunofluorescence staining, cells were seeded on coverslips and after 24 h, the cells were treated as indicated. After 48 h, cells were fixed with 4% (w/v) paraformaldehyde for 10 min and permeabilized with 0.5% Triton X-100 for 10 min. After blocking with 3% BSA in PBS, the cells were stained with anti-4-hydroxynonenal polyclonal antibody (ab48506; Abcam, Cambridge, United Kingdom) or ALDH7A1 antibody (ab53278; Abcam, Cambridge, United Kingdom) overnight at 4 °C and then in Alexa Fluor 594-conjugated anti-mouse antibody (A11032; Life Technologies, Carlsbad, CA, USA) or Alexa Fluor 488-conjugated anti-rabbit antibody (A21206; Life Technologies, Carlsbad, CA, USA) diluted in 3% BSA in PBS for 1h at room temperature in dark. The cells were then stained with Hoechst 33342 (H1399, Thermo Fisher Scientific, Waltham, MA, USA) 10 min in PBS to visualize nuclei. The samples were examined under a Zeiss LSM780 confocal microscope (Carl Zeiss, Oberkochen, Baden-Württemberg, Germany).

### Relative quantitation of metabolites using liquid chromatography-tandem mass spectrometry (LC-MS/MS)

Using LC-MS/MS equipped with 1290 HPLC (Agilent, Santa Clara, CA, USA), Qtrap 5500 (ABSciex, Concord, Ontario, Canada), and reverse phase (Synergi fusion RP 50 × 2 mm) columns.

### Relative quantitation of fatty acyl CoA using LC-MS/MS

#### Sample preparation

One million cells were harvested using 1.4 ml of cold methanol/H_2_O (80/20, v/v) after sequential washing with PBS and H_2_O. Cells were lysed by vigorous vortexing prior to addition of 100 μl internal standard (Malonyl-^13^C_3_ CoA; 5 μM). Chloroform was added and metabolites were extracted from the aqueous phase by liquid-liquid extraction. The aqueous phase was dried in a vacuum centrifuge and the sample was reconstituted with 50 μl of H_2_O/MeOH (50/50 v/v) prior to LC-MS/MS analysis.

#### LC-MS/MS

Fatty acyl CoA was analyzed by LC-MS/MS equipped with 1290 HPLC (Agilent), Qtrap 5500 (ABSciex), and reverse phase (Zorbax 300Extend-C18 2.1 × 150 mm) columns. Next, 3 μl sample was injected into the LC-MS/MS system and ionized by a turbo spray ionization source. Acetonitrile/H_2_O (10/90) with 15 mM ammonium hydroxide and acetonitrile with 15 mM ammonium hydroxide were used as mobile phase A and B, respectively. The separation gradient was as follows: hold at 0% B for 3 min; 0% to 50% B for 2 min; 50% to 70% B for 5 min; 70% to 0% B for 0.1 min; hold at 0% B for 4.9 min. LC flow was 200 μl/min and the column temperature was kept at 25 °C. Multiple reaction monitoring was used in positive ion mode and the extracted ion chromatogram corresponding to the specific transition for each fatty acyl CoA was used for quantitation. The calibration range for fatty acyl CoAs was 0.1-10000 nM (r^2^≥0.99). Data analysis was performed using Analyst 1.5.2 software.

### Pancreatic cancer patients' data analysis

The expression level of ALDH isoforms in PAAD compared with matched normal was analyzed by GEPIA webserver (http://gepia.cancer-pku.cn/). Tumor data were form TCGA (n = 179), normal data were from both TCGA and GTEx (n = 171). The relationship between ALDH isoforms expression level and prognosis of PAAD patients was analyzed by cBioPortal (www. cbioportal.org) using a pancreatic adenocarcinoma (TCGA, Provisional) dataset. ALDH isoforms expression level less than standard deviation from the mean value was considered as low.

### Sulforhodamine B assay: cell growth assay

To measure cell proliferation, we used SRB assay 45. Assay method was followed as we published before 46.

### Fatty acid oxidation assay

To assess the ability to oxidize exogenous fatty acids, the oxygen-consumption rate (OCR) of cells was analyzed using the XFe96 extracellular flux analyzer (Seahorse Bioscience, North Billerica, Massachusetts, USA). Cells were transfected with NT siRNA or ALDH7A1 siRNA (40 nM) in 60 mm dishes. After 48 h, transfected cells were seeded in XF cell culture microplate at a cellular density of 30,000/well with substrate-limited medium (XF Assay Medium Modified DMEM (Seahorse Bioscience, North Billerica, Massachusetts, USA) with 0.5 mM glucose, 1x GlutaMAX (Thermo Fisher Scientific, Waltham, MA, USA), 0.5 mM carnitine (Sigma-Aldrich, St. Louis, MO, USA) and 1% FBS and incubated for 24 h at 37 °C. To test the effect of gossypol on cellular respiration, 30,000 cells were plated in each well of a seahorse microplate and next day these cells were treated with 5 μM gossypol in substrate-limited medium for 24 h. The next day, the medium was changed to FAO assay medium (111 mM NaCl, 4.7 mM KCl, 1.25 mM CaCl_2_, 2.0 mM MgSO_4_, 1.2 mM Na_2_HPO_4_, 2.5 mM glucose, 0.5 mM carnitine and 5 mM HEPES) for 45 min. Palmitate-BSA (200 µM palmitate conjugated with 34 µM BSA) or BSA (34 µM) (Seahorse Bioscience, North Billerica, Massachusetts, USA) were added and the OCR analyzed. The samples were mixed (3 min) and measured (3 min) using XFe96 extracellular flux analyzer (Seahorse Bioscience, North Billerica, Massachusetts, USA). ATP synthase inhibitor oligomycin (1.6 µM), the chemical uncoupler FCCP (0.8 µM), and the electron transport inhibitor rotenone/antimycin A (0.5 µM) dissolved in FAO assay medium were injected at the indicated time points. Oligomycin, FCCP, rotenone, and antimycin A are purchased from Agilent (103015-100; Agilent, Santa Clara, CA, USA). Raw data were normalized by SRB assay.

### Preclinical xenograft tumor models

Balb/c-nu/nu mice (Orient, Seoul, Korea) were aged between 6 and 8 weeks before tumor induction. This study was reviewed and approved by the Institutional Animal Care and Use Committee (IACUC) of the National Cancer Center Research Institute, which is an Association for Assessment and Accreditation of Laboratory Animal Care International (AAALAC International) accredited facility that abides by the Institute of Laboratory Animal Resources guide (protocols: NCC-16-345, NCC-19-460). MIA PaCa-2 cells (1 × 10^7^) and AsPC-1 cells (1 × 10^7^) in 100 μl PBS were subcutaneously inoculated using a 1-ml syringe. After a week, the mice were divided into four groups: a control group treated with vehicle only, gossypol or phenformin single, and combination of gossypol and phenformin. Vehicle (5% DMSO and 5% cremophor in PBS, 100 μl) alone, gossypol (80 mg/kg/100 μl), and phenformin (100 mg/kg/100 μl) were administered orally once per day, 6 days/week for 7~9 weeks (n = 5 or 6). Primary tumor size was measured every week using calipers. Tumor volume was calculated using the formula, V = (A × B^2^)/2, where V is the volume (mm^3^), A is the long diameter, and B is the short diameter.

### *Aldh7a1* knockout and PDAC mice

*Aldh7a1* knockout has been generated using CRISPR/Cas9 and zygote electroporation. Mixture of Cas9 protein (100 ng/µl) and gRNA (50 ng/µl) was transferred to mouse embryo using zygote electroporation 47. Target sequences in exon 5 of small guide RNA (sgRNA) were selected using CRISPR design tool (crispor.tefor.net): 5'-ATT ATG CTG CTG GCT TGT CG-3' and 5'-AGG CGA GGT TCA GGA GTA CG -3'. Indel mutations in F1 mice were identified after TA cloning and Sanger sequencing. Of mutant mice, the mice having 38-nt deletion was bred with KPC mice. Cas9 protein (EnGen Cas9 NLS) was purchased from NEB. sgRNAs were generated using MEGAshortscript T7 transcription kit (ThermoFisher).

*Pdx1-Cre* mice, *Kras^G12D^* mice and *Trp53^R172H^* mice were obtained from NCI mouse repository (http://mouse.ncifcrf.gov). *Kras^G12D^; Pdx1-Cre* (KC) mice was achieved by crossing *Kras^G12D^* mice with *Pdx1-Cre* mice. *Kras^G12D^; Trp53^ R172H^; Pdx1-Cre* (KPC) mice were obtained by crossing KC mice with *Trp53^ R172H^* mice. This study was reviewed and approved by the Institutional Animal Care and Use Committee (IACUC) of the National Cancer Center Research Institute, which is an Association for Assessment and Accreditation of Laboratory Animal Care International (AAALAC International) accredited facility that abides by the Institute of Laboratory Animal Resources guide (protocols: NCC-19-459, NCC-19-462, NCC-19-506). The sequences of primer are listed in Table S4.

### Clonogenic assay: cell growth assay

Cells were plated in 6 well plates at (1000-5000) cells per well in 2 ml media. Media was not changed throughout the course of the experiment. After 14 days, colonies were stained with 0.005% crystal violet staining solution.

### Measurement of mitochondrial membrane potential (∆ψm)

Cells were cultured into 100 mm dishes and treated with Gossypol 5 μM, Phenformin 100 μM alone or both for 24 h. The tetramethylrhodamine-ethylester (TMRE; 87917; Sigma-Aldrich, St. Louis, MO, USA) assay was performed, according to the manufacturer's protocol and analyzed by FACS Calibur flow cytometry (BD Falcon, Bedford, MA, USA).

### Immunoblot analysis

Harvested cells were lysed with RIPA cell lysis buffer in the presence of protease and phosphatase inhibitor cocktail (Sigma, St. Louis, MO, USA). The protein concentration of the cell lysates was quantified by a BCA Pierce Protein Assay Kit (Thermo Fisher Scientific, Waltham, MA, USA). The same amount of protein samples was loaded onto 10% SDS-PAGE and transferred onto PVDF membranes. After blocking by 5% BSA, the membranes were incubated in the primary antibodies diluted in 5% BSA buffer for overnight at 4 °C and then in the HRP-conjugated secondary antibody for 1 h at room temperature. The protein band images were captured with ECL reagent (Thermo Fisher Scientific, Waltham, MA, USA). The primary antibodies used in the experiments were ALDH1L1 (ab175198, Abcam, Cambridge, UK), ALDH1A3 (ab129815, Abcam), ALDH1L2 (ab113496, Abcam), ALDH2 (ab108306, Abcam), ALDH3A1 (ab76976, Abcam), ALDH3B1 (SAB4500866, Sigma-Aldrich), ALDH4A1 (ab185208, Abcam), ALDH7A1 (ab53278, Abcam), Flag (F1804, Sigma-Aldrich) and β-actin (sc-47778, Santa Cruz Biotechnology).

### Immunohistochemistry

Formaldehyde (4%) fixed specimens were paraffin-embedded and cut at a thickness of 4 μm. Sections were dried for 1h at 56 °C and immunohistochemical staining performed with the automated instrument Discovery XT (Ventana medical system, Tucson Arizona, USA) using the Chromomap DAB Detection kit as follow: sections were deparaffinized and rehydrated with EZ prep (Ventana) and washed with Reaction buffer (Venatana). The antigens were retrieved with heat treatment in pH 6.0 Citrate buffer (Ribo CC, ventana) at 90 °C for 30 min for anti-Ki-67 (ab15580; Abcam, Cambridge, United Kingdom), CK-19 (ab52625, Abcam, Cambridge, UK), α-SMA (ab5694, Abcam), 4-HNE (ab46545, Abcam) and ALDH7A1 (ab53278, Abcam).

### XF cell mito stress analysis

Cells were treated with the indicated drug for 24 h. For OCR determination, cells were incubated in XF base medium supplemented with 10 mM glucose, 1 mM sodium pyruvate, and 2 mM L-glutamine, and were equilibrated in a non-CO_2_ incubator for 1 h before starting the assay. The samples were mixed (3 min) and measured (3 min) using the XFe96 extracellular flux analyzer (Seahorse Bioscience, North Billerica, MA, USA). Oligomycin (0.75 µM), FCCP (1 µM), and rotenone/antimycin A (0.5 µM) were injected at the indicated time points. Finally, the OCR was normalized using the SRB assay.

### TUNEL assay: cell death detection

A fluorometric TUNEL detection kit was used according to the manufacturer's instructions (11684795910; Roche Applied Science, Indianapolis, IN). In brief, cells were treated with 5 µM gossypol or 100 µM phenformin single and combination of gossypol and phenformin for 48 h and fixed with 4% paraformaldehyde in PBS for 10 min, permeabilized with 0.5% Triton X-100 in PBS at 4 °C for 2 min, and incubated with the provided fluorescein-conjugated TUNEL reaction mixture in a humidified chamber at 37 °C for 1 h in dark. Omission of the addition TdT enzyme in the TUNEL reaction mixture was included as negative control. The cells were then mounted with 4',6-diamidino-2-phenylindole (DAPI) mounting medium to visualize nuclei (Vectashield mounting medium; Vector Laboratories, Burlingame, CA). TUNEL- and DAPI-stained nuclei staining were examined under a Zeiss LSM780 confocal microscope (Carl Zeiss, Oberkochen, Baden-Württemberg, Germany).

### Tissue array from clinical samples

Tissue microarrays (TMA) of PDAC patients were purchased (HPan-Ade120Sur-01 PDAC TMA: US Biomax, Bethesda, MD, USA). The TMA of PDAC consists of 63 cases of pancreatic ductal adenocarcinoma. Immunohistochemical staining of ALDH7A1 was performed according to standard procedures. Immunostained TMAs were scanned using phenochart (Perkin Elmer, Waltham, MA, USA). Staining pattern and intensity of ALDH7A1 were analyzed by special pathologist (E.H.), which showed reasonable consistency (intraclass correlation coefficient = 0.955). The staining intensity was divided into negative, weak, moderate, and strong according to the ALDH7A1 abundance. Overall survival was analyzed statistically by Logrank value tests using GraphPad PRISM 5 (GraphPad Software, San Diego, CA, USA).

### Statistical analysis

Statistical analysis was performed using the Student's t test as appropriate. Tumor growth and tumor weight was analyzed statistically by two-way analysis of variance (ANOVA) tests using GraphPad PRISM 5 (GraphPad Software, San Diego, CA, USA).

## Supplementary Material

Supplementary figures and tables.

## Abbreviations

PDAC: pancreatic ductal adenocarcinoma
NADH: nicotinamide adenine dinucleotide
ATP: adenosine tri-phosphate
TCA cycle: tricarboxylic acid cycle (also known as the Krebs cycle)
FAO: fatty acid oxidation
KPC mouse: *Kras^G12D^*, *Trp53^R172H^*, *Pdx1-Cre* mouse
CoA: coenzyme A
4-HNE: 4-Hydroxynonenal
OxPhos: Oxidative phosphorylation
ALDH7A1: protein name of human or mouse aldehyde dehydrogenase 7A1
*ALDH7A1*: gene name of human ALDH7A1
*Aldh7a1*: gene name of mouse ALDH7A1
*KRAS*: gene name of human KRAS
*Kras*: gene name of mouse KRAS
