# Supplementary material for: Overall survival of pancreatic ductal adenocarcinoma is doubled by Aldh7a1 deletion in the KPC mouse
Source: Theranostics. 2021 Jan 19;11(7):3472–88. doi: 10.7150/thno.53935 (PMC7847681; doi:10.7150/thno.53935)
Supplement: Supplementary file 1 — Supplementary figures and tables. [file thnov11p3472s1.pdf]

Figure S1

A

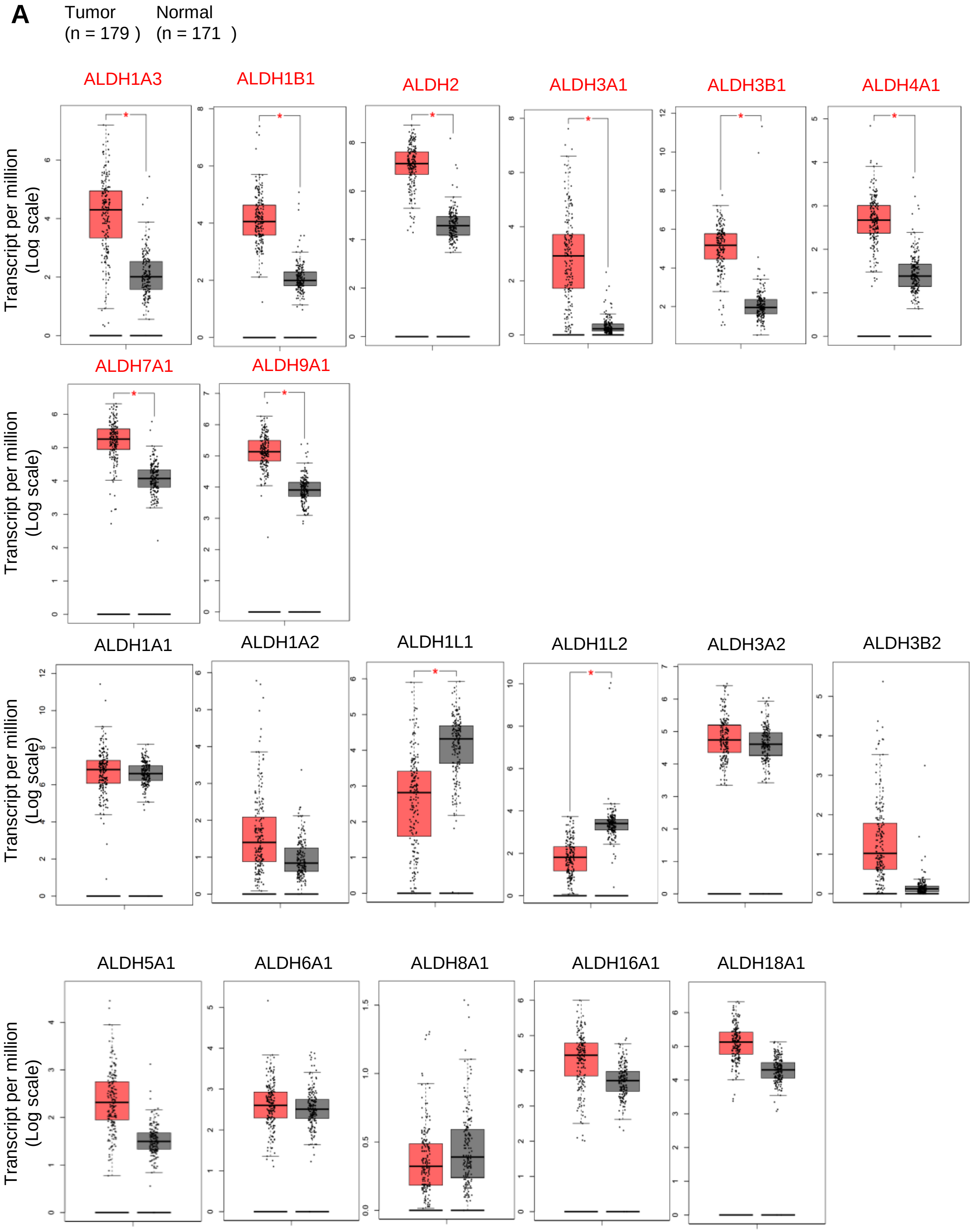
Tumor
(n = 179)
Normal
(n = 171)
ALDH1A3
ALDH1B1
ALDH2
ALDH3A1
ALDH3B1
ALDH4A1
ALDH7A1
ALDH9A1
ALDH1A1
ALDH1A2
ALDH1L1
ALDH1L2
ALDH3A2
ALDH3B2
ALDH5A1
ALDH6A1
ALDH8A1
ALDH16A1
ALDH18A1
Transcript per million
(Log scale)

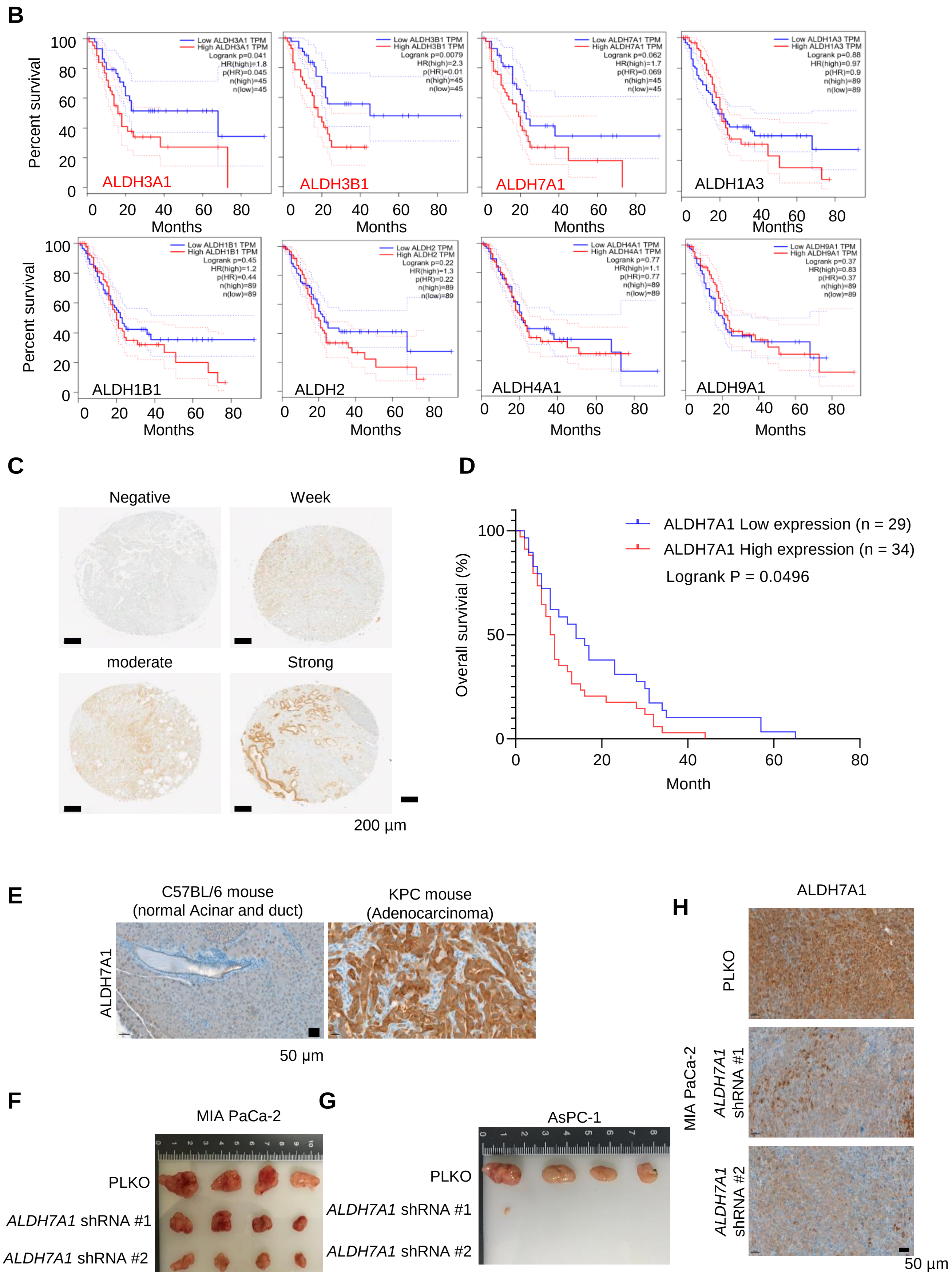
B
Percent survival
Months
ALDH3A1
ALDH3B1
ALDH7A1
ALDH1A3
ALDH1B1
ALDH2
ALDH4A1
ALDH9A1
Low ALDH3A1 TPM
High ALDH3A1 TPM
Logrank p=0.041
HR(high)=1.8
p(HR)=0.045
n(high)=45
n(low)=45
Low ALDH3B1 TPM
High ALDH3B1 TPM
Logrank p=0.0079
HR(high)=2.3
p(HR)=0.01
n(high)=45
n(low)=45
Low ALDH7A1 TPM
High ALDH7A1 TPM
Logrank p=0.062
HR(high)=1.7
p(HR)=0.069
n(high)=45
n(low)=45
Low ALDH1A3 TPM
High ALDH1A3 TPM
Logrank p=0.88
HR(high)=0.97
p(HR)=0.9
n(high)=89
n(low)=89
Low ALDH1B1 TPM
High ALDH1B1 TPM
Logrank p=0.45
HR(high)=1.2
p(HR)=0.44
n(high)=89
n(low)=89
Low ALDH2 TPM
High ALDH2 TPM
Logrank p=0.22
HR(high)=1.3
p(HR)=0.22
n(high)=89
n(low)=89
Low ALDH4A1 TPM
High ALDH4A1 TPM
Logrank p=0.77
HR(high)=1.1
p(HR)=0.77
n(high)=89
n(low)=89
Low ALDH9A1 TPM
High ALDH9A1 TPM
Logrank p=0.37
HR(high)=0.83
p(HR)=0.37
n(high)=89
n(low)=89
C
Negative
Week
moderate
Strong
200 µm
D
Overall survival (%)
Month
ALDH7A1 Low expression (n = 29)
ALDH7A1 High expression (n = 34)
Logrank P = 0.0496
E
C57BL/6 mouse
(normal Acinar and duct)
KPC mouse
(Adenocarcinoma)
ALDH7A1
50 µm
F
MIA PaCa-2
PLKO
ALDH7A1 shRNA #1
ALDH7A1 shRNA #2
G
AsPC-1
PLKO
ALDH7A1 shRNA #1
ALDH7A1 shRNA #2
H
ALDH7A1
MIA PaCa-2
PLKO
ALDH7A1 shRNA #1
ALDH7A1 shRNA #2
50 µm

Figure S2

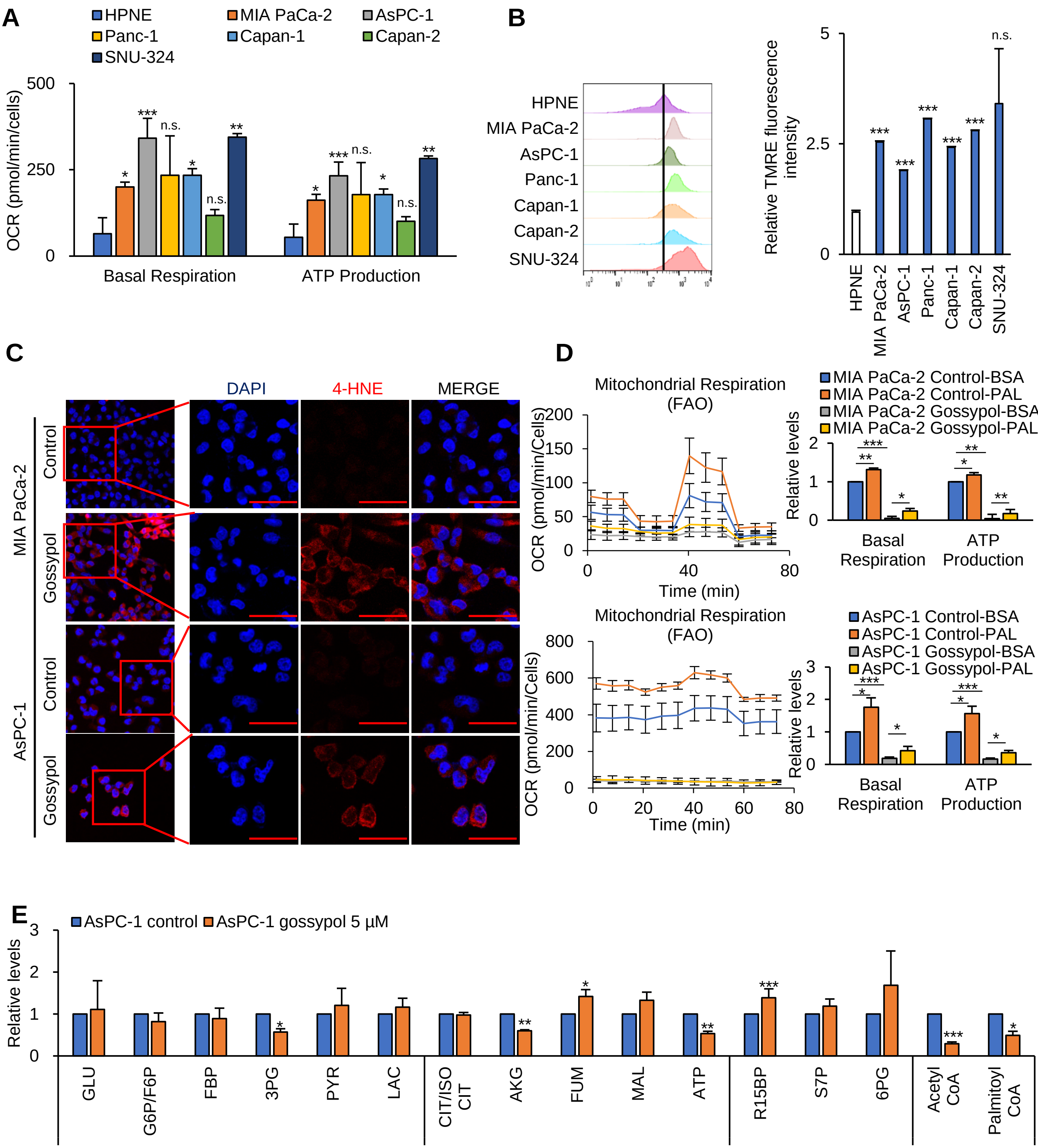

A
HPNE
MIA PaCa-2
AsPC-1
Panc-1
Capan-1
Capan-2
SNU-324
OCR (pmol/min/cells)
Basal Respiration
ATP Production
n.s.
B
Relative TMRE fluorescence intensity
C
DAPI
4-HNE
MERGE
MIA PaCa-2
AsPC-1
Control
Gossypol
D
Mitochondrial Respiration (FAO)
OCR (pmol/min/Cells)
Time (min)
MIA PaCa-2 Control-BSA
MIA PaCa-2 Control-PAL
MIA PaCa-2 Gossypol-BSA
MIA PaCa-2 Gossypol-PAL
Relative levels
AsPC-1 Control-BSA
AsPC-1 Control-PAL
AsPC-1 Gossypol-BSA
AsPC-1 Gossypol-PAL
E
AsPC-1 control
AsPC-1 gossypol 5 μM
GLU
G6P/F6P
FBP
3PG
PYR
LAC
CIT/ISO CIT
AKG
FUM
MAL
ATP
R15BP
S7P
6PG
Acetyl CoA
Palmitoyl CoA

Figure S3

A

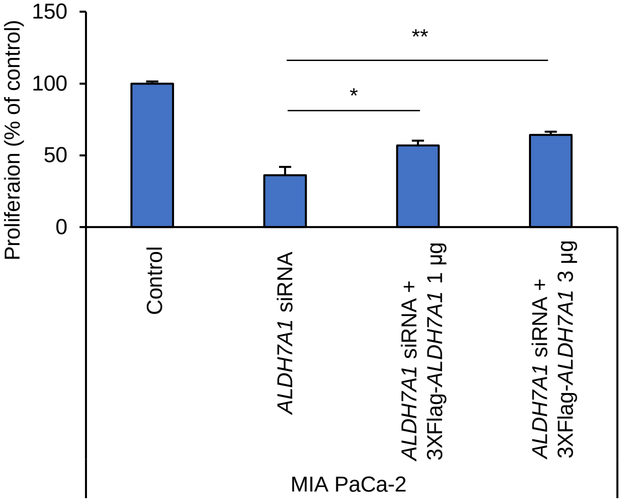

B

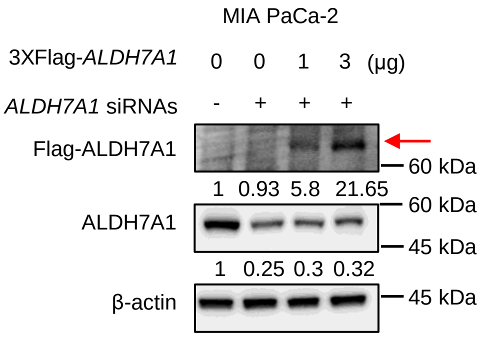

C

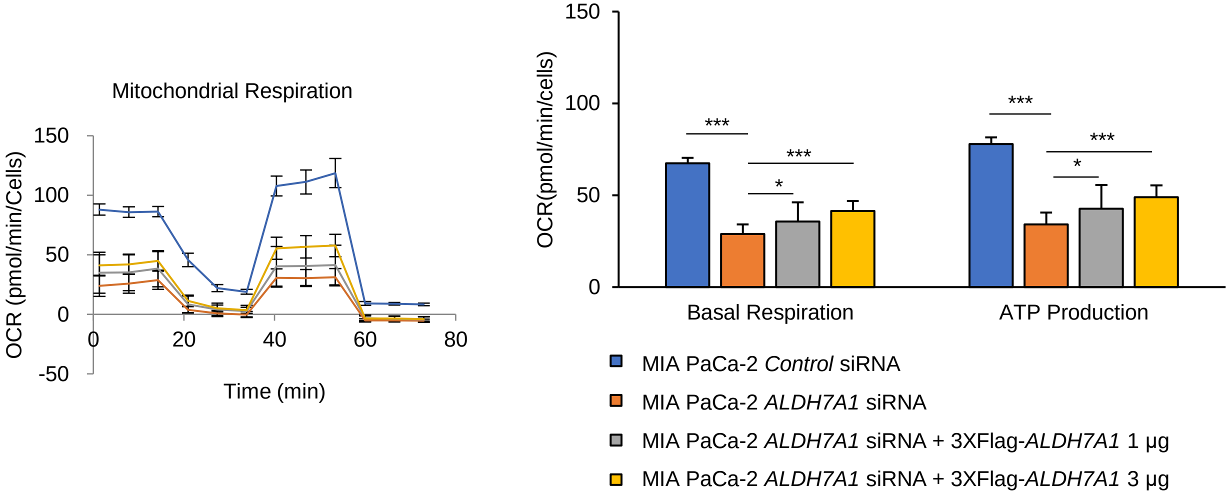

Figure S4

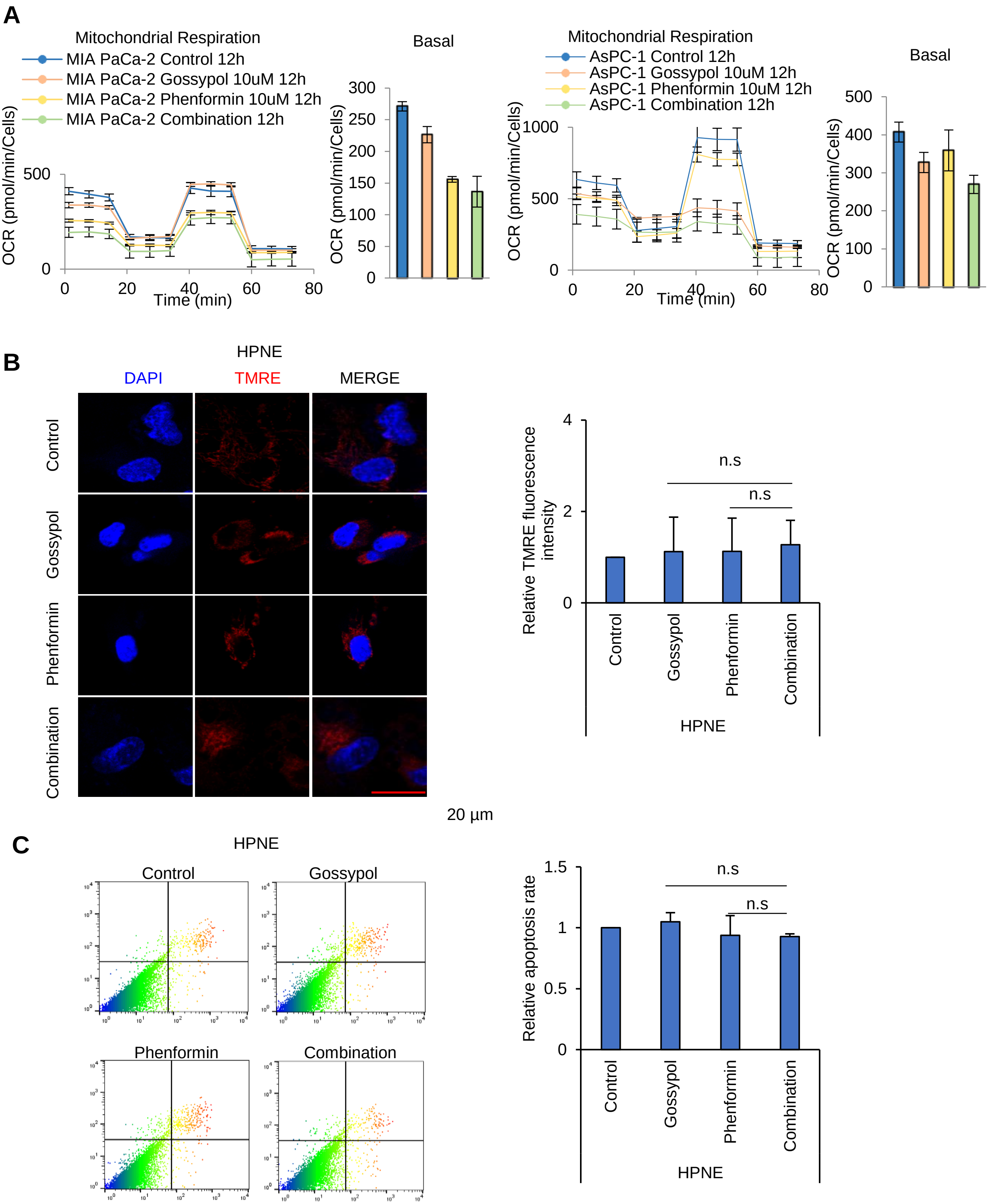

A
Mitochondrial Respiration
MIA PaCa-2 Control 12h
MIA PaCa-2 Gossypol 10uM 12h
MIA PaCa-2 Phenformin 10uM 12h
MIA PaCa-2 Combination 12h
OCR (pmol/min/Cells)
Time (min)
Basal
Mitochondrial Respiration
AsPC-1 Control 12h
AsPC-1 Gossypol 10uM 12h
AsPC-1 Phenformin 10uM 12h
AsPC-1 Combination 12h
B
HPNE
DAPI
TMRE
MERGE
Control
Gossypol
Phenformin
Combination
20 µm
Relative TMRE fluorescence intensity
n.s
n.s
HPNE
C
HPNE
Control
Gossypol
Phenformin
Combination
Relative apoptosis rate
n.s
n.s
HPNE

Figure S5

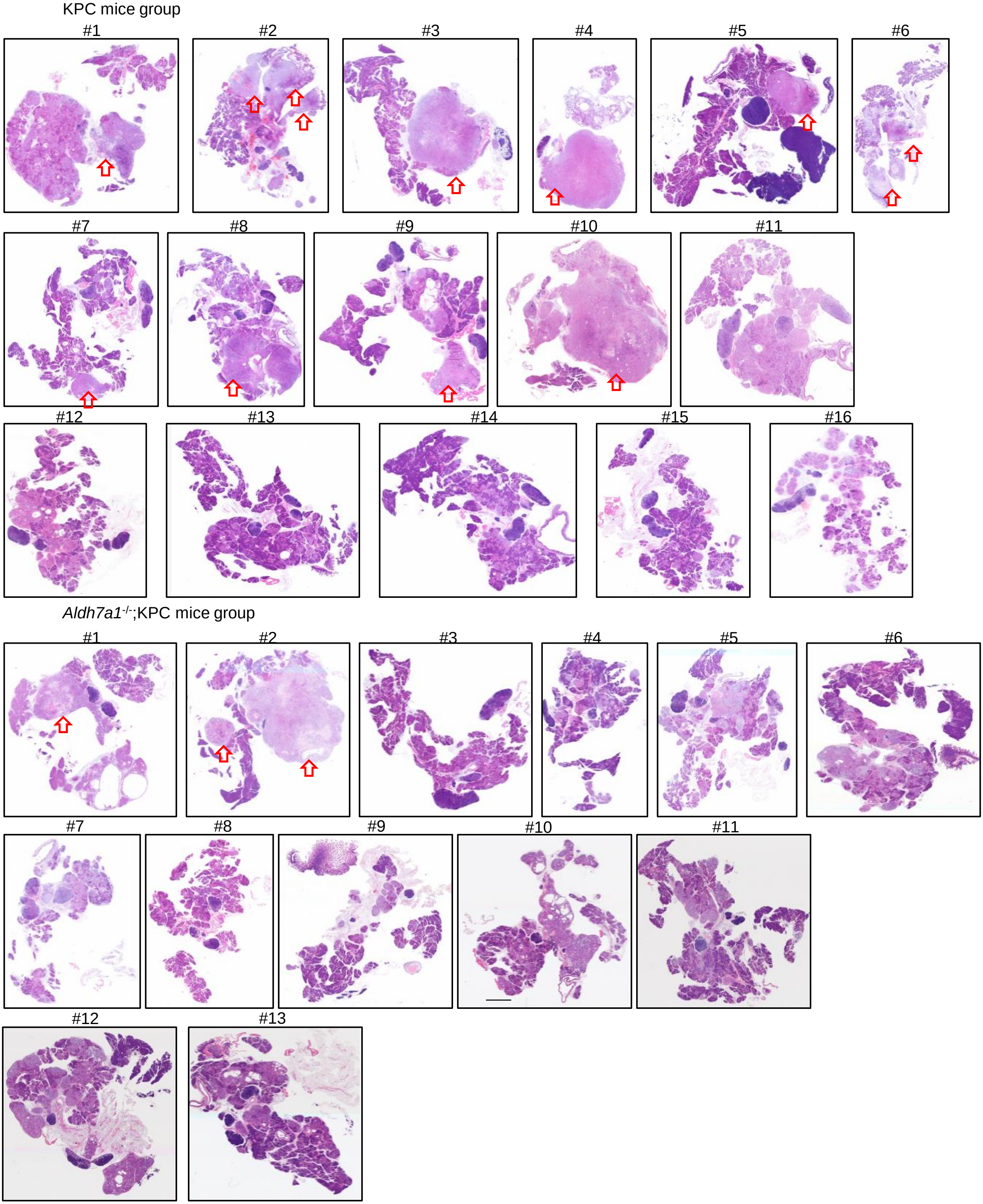

KPC mice group
#1
#2
#3
#4
#5
#6
#7
#8
#9
#10
#11
#12
#13
#14
#15
#16
Aldh7a1-/-;KPC mice group
#1
#2
#3
#4
#5
#6
#7
#8
#9
#10
#11
#12
#13

Figure S6

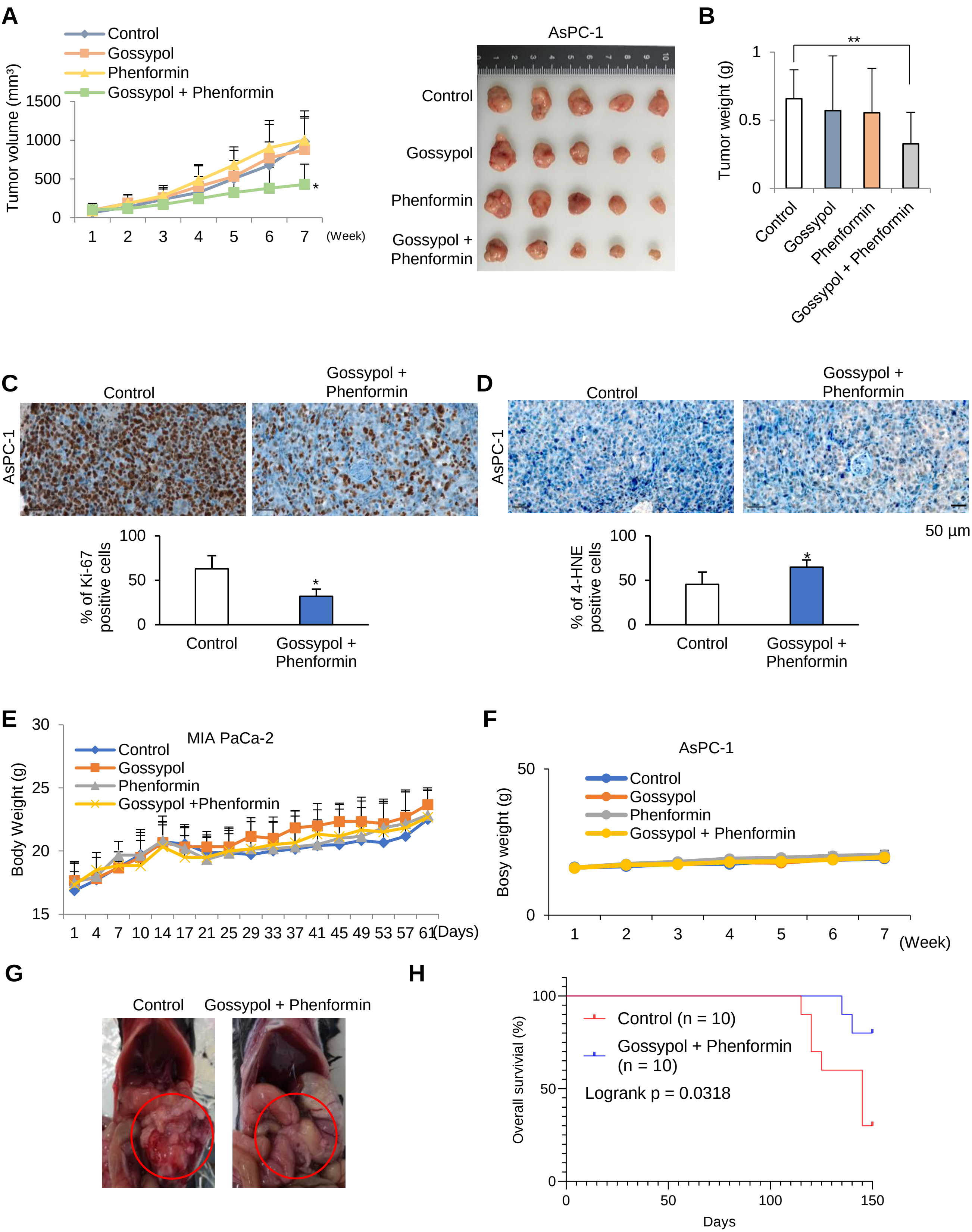
A
Control
Gossypol
Phenformin
Gossypol + Phenformin
Tumor volume (mm³)
1500
1000
500
0
1 2 3 4 5 6 7
(Week)
*
AsPC-1
Control
Gossypol
Phenformin
Gossypol +
Phenformin
B
Tumor weight (g)
1
0.5
0
**
Control
Gossypol
Phenformin
Gossypol + Phenformin
C
Control
Gossypol +
Phenformin
AsPC-1
% of Ki-67
positive cells
100
50
0
*
Control
Gossypol +
Phenformin
D
Control
Gossypol +
Phenformin
AsPC-1
50 μm
% of 4-HNE
positive cells
100
50
0
*
Control
Gossypol +
Phenformin
E
MIA PaCa-2
Control
Gossypol
Phenformin
Gossypol +Phenformin
Body Weight (g)
30
25
20
15
1 4 7 10 14 17 21 25 29 33 37 41 45 49 53 57 61
(Days)
F
AsPC-1
Control
Gossypol
Phenformin
Gossypol + Phenformin
Bosy weight (g)
50
0
1 2 3 4 5 6 7
(Week)
G
Control
Gossypol + Phenformin
H
Overall survivial (%)
100
50
0
Control (n = 10)
Gossypol + Phenformin
(n = 10)
Logrank p = 0.0318
0 50 100 150
Days

Figure S7

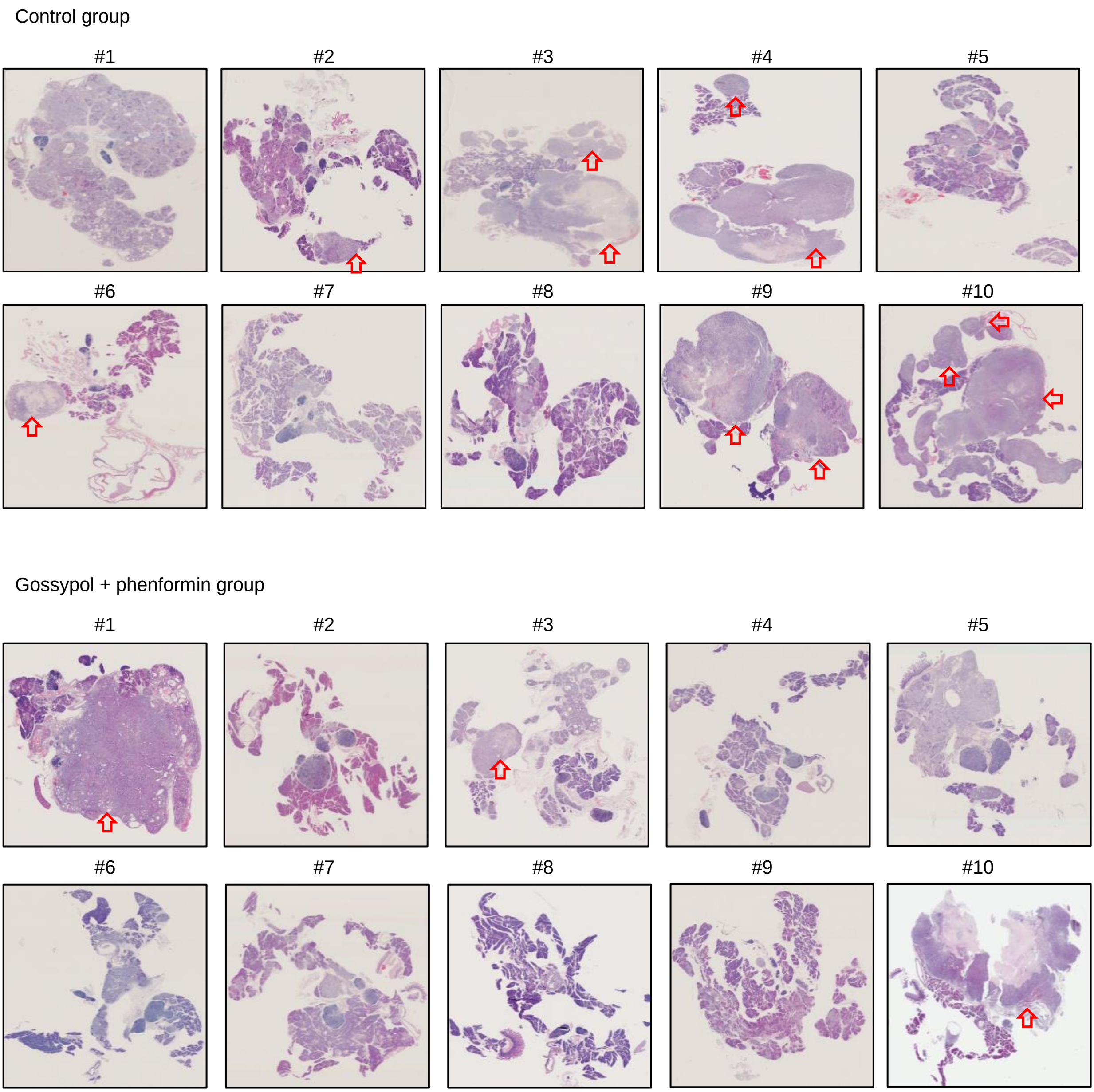

Control group
#1
#2
#3
#4
#5
#6
#7
#8
#9
#10
Gossypol + phenformin group
#1
#2
#3
#4
#5
#6
#7
#8
#9
#10

Table S1. The sequence of siRNAs

Gene name	Sequence	
	sense（5'-3'）	antisense（5'-3'）
ALDH7A1 #1	GGAAAUUAUGUAGAACCGAdTdT	UCGGUUCUACAUAAUUUCCdTdT
ALDH7A1 #2	GGAUGAUUGGAGGACCUAUdTdT	AUAGGUCCUCCAAUCAUCCdTdT
Negetive control	UUCUCCGAACGUGUCACGUTT	ACGUGACACGUUCGGAGAATT

Table S2. Overexpression primer of ALDH7A1

Vector	Gene template	Primer sequences	Enzyme site	Amplicon size (bp)
p3XFlag-CMV	*ALDH7A1*	F': TGACGATGACAAGCTT ATGTGGCGCCTTCCTCGC	Hind III	1620
		R': TCGCGGCCGCAAGCTT TTACTGAAACTTGATTCC	Hind III	

Table S3. The sequence of shRNAs

Gene name	Sequence
ALDH7A1 #1 (TRCN0000028447)	CCGGCGGGAGAAGATCCAAGTACTACTCGAGTAGTACTTGGATCT TCTCCCGTTTTT
ALDH7A1 #2 (TRCN0000028463)	CCGGGCTCCGATTCTCTATGTCTTTCTCGAGAAAGACATAGAGAAT CGGAGCTTTTT
ALDH7A1 #3 (TRCN0000028401)	CCGGCCAAGGAATCAAGTTTCAGTACTCGAGTACTGAAACTTGATT CCTTGGTTTTT
ALDH7A1 #4 (TRCN0000028408)	CCGGGCACTGATTGAGCAGTGGAATCTCGAGATTCCACTGCTCAA TCAGTGCTTTTT

Table S4. The sequence of primers

Gene name	Sequence
ALDH7A1	Forward: AGG TGT CTT TGG AGA TGG GGA A
	Reverse: GC GTT TGA AGC TCT GTG TCG
LSL Kras	Wild type forward: TGT CTT TCC CCA GCA CAG T
	Reverse: CTG CAT AGT ACG CTA TAC CCT GT
	Mutant forward: GCA GGT CGA GGG ACC TAA TA
Pdx1-Cre	Forward: CTG GAC TAC ATC TTG AGT TGC
	Reverse: TTC TTG CGA ACC TCA TCA CTC GTT G
Trp53	Wild type forward: AGG TGT GGC TTC TGG CTT C
	Reverse: GAA ACT TTT CAC AAG AAC CAG ATC A
	Mutant forward: CCA TGG CTT GAG TAA GTC TGC A

Supplementary Figure 1. Pancreatic cancer patients with high ALDH7A1 expression showed poor prognosis (A) ALDH isoforms expression level in PAAD patients was compared with matched normal by GEPIA webserver (http://gepia.cancer-pku.cn/). ALDH isoforms significantly overexpressed in PAAD were colored red. **(B)** For the ALDH isoforms significantly overexpressed in PAAD, the correlation with patient's prognosis were analyzed by GEPIA webserver. Cutoff-Low 25% versus Cutoff-High 75% were analyzed. The isoforms associated with patients' poor prognosis were colored red. **(C)** representative images of TMA stained for ALDH7A1 showing ALDH7A1-negative pancreatic cancer (5 of 63), ALDH7A1-week positive pancreatic cancer (24 of 63), ALDH7A1-moderate positive pancreatic cancer (23 of 63) and ALDH7A1-strong positive pancreatic cancer (11 of 63). **(D)** The overall survival of 64 patients with pancreatic cancer in ALDH7A1 low-score (negative and week) and high-score groups (moderate and strong). **(E)** The immunohistochemical staining of ALDH7A1 showed significant increase of ALDH7A1 in pancreas of KPC mouse to compare to pancreas of normal mouse. **(F)** Representative images of tumors derived from ALDH7A1 shRNA transduced MIA PaCa-2 cells. **(G)** Representative images of tumors derived from ALDH7A1 shRNA transduced AsPC-1 cells. **(H)** Immunohistochemistry of ALDH7A1 in MIA PaCa-2 cancer cell lines showed significant reduction of ALDH7A1 by ALDH7A1 shRNA. Scale bar =50 μm.

Supplementary Figure 2. OxPhos was increased in pancreatic cancer cells compared to pancreas normal duct cell. (A) Oxygen consumption rate was measured in pancreatic cancer cells compared to normal cell by Seahorse XF analyzer. **(B)** Mitochondria membrane potential was measured in pancreatic cancer cells compared to normal cell by TMRE assay. **(C)** Treatment of gossypol increased 4-hydroxynonenal level in Pancreatic cancer cells as determined by Immunocytochemistry analysis. Scale bar = 50 μm. **(D)** Seahorse XF analysis of cells treated sequentially with oligomycin, the chemical uncoupler FCCP and antimycin A (downward arrows) in the presence of bovine serum albumin alone (BSA) or palmitate-BSA. Cells with treated gossypol showed decreased fatty acid oxidation compared to control cells. **(E)** Effect of gossypol treatment (5 μM) on metabolites derived from various metabolic pathways in AsPC-1 cells. Relative pool sizes of metabolites after ALDH7A1 siRNA treatment for 48 h or gossypol treatment for 24 h were assessed by targeted LC-MS/MS. Data are expressed as the mean and standard deviation of three independent experiments. *$p < 0.05$, **$p < 0.01$, ***$p < 0.001$.

Supplementary Figure 3. *ALDH7A1* re-expression recovered cell proliferation and oxygen consumption rate. (A) Treatment of *ALDH7A1* siRNA for 72 h showed inhibition of cell growth in MIA PaC-2. And *ALDH7A1* re-expression for 48 h showed recovery of cell growth as determined by SRB assay. (B) Western blot analysis of MIA PaCa-2 cancer cell lines demonstrating their ALDH7A1 and Flag status. Actin used as a loading control. (C) Re-expression of ALDH7A1 recovered oxygen consumption rates (OCR) and ATP production as determined by Seahorse XFe analyzer. Data are expressed as the mean and standard deviation of three independent experiments. *p < 0.05, **p < 0.01, ***p < 0.001.

Supplementary Figure 4. Gossypol treatment combined with phenformin does not affect mitochondrial membrane potential and cell death of HPNE. (A) Combined treatment of 10 μM gossypol and 10 μM phenformin after 12 h synergistically reduced Oxygen Consumption Rates and respiration parameters as determined by Seahorse XFe analyzer (B) Combined treatment for 24 h not reduced the mitochondrial membrane potential in HPNE cells, as determined by TMRE staining and live cell imaging. Scale bar = 20 μm. (C) Treatment of 5 μM gossypol combined with 100 μM phenformin for 24 h on HPNE cells not led to cell death (determined in Annexin v assay). Data are expressed as the mean and standard deviation of three independent experiments. *p < 0.05, **p < 0.01, ***p < 0.001.

Supplementary Figure 5. Representative H&E of pancreas from KPC mouse and *Aldh7a1*$^{-/-}$KPC mouse. Red arrow is Pancreatic Ductal adenocarcinoma.

Supplementary Figure 6. Gossypol treatment combined with phenformin synergistically suppresses tumor growth in a human pancreatic cancer xenograft mouse model. (A) AsPC-1 (1×10^7) cells were injected in 6–8-week-old BALB/c nude mice. When the volume of the tumor mass reached 100 mm^3, the mice were randomly assigned to one of four treatment groups including vehicle control, gossypol, phenformin, and combination of gossypol and phenformin (n=5 per group). Gossypol (80 mg/kg body weight), phenformin (100 mg/kg body weight), and vehicle were administered orally 6 days/week. Graph (left) and photograph (right) shows a synergistic decrease in tumor growth after combined treatment of gossypol and phenformin as measured using calipers. (B) Final weight of subcutaneous tumors derived from AsPC-1. (C) IHC analysis of Ki67 staining in and AsPC-1 tumor xenograft tissues. (D) IHC analysis of 4-HNE staining in and AsPC-1 tumor xenograft tissues. (E) Body weight of mice was measured after inoculation of MIA PaCa-2. (F) Body weight of mice was measured weekly after inoculation of AsPC-1. (G) Gross morphology pictures of a primary tumor in KPC mouse treated with vehicle or gossypol combined with phenformin. (H) Kaplan-Meier survival curves of KPC mouse treated with vehicle or gossypol combined with phenformin. *$p < 0.05$, **$p < 0.01$, ***$p < 0.001$.

Supplementary Figure 7. Representative H&E of pancreas from control mouse and gossypol combined with phenformin mouse. Red arrow is Pancreatic Ductal adenocarcinoma.

Supplementary Figure 8. Representative H&E of pancreas from control mouse and gossypol combined with phenformin mouse. Red arrow is Pancreatic Ductal adenocarcinoma.
